# Multimodal imaging of a humanized orthotopic model of hepatocellular carcinoma in immunodeficient mice

**DOI:** 10.1038/srep35230

**Published:** 2016-10-14

**Authors:** Tao Wu, Emilie Heuillard, Véronique Lindner, Ghina Bou About, Mihaela Ignat, Jean-Philippe Dillenseger, Nicolas Anton, Eugénie Dalimier, Francine Gossé, Gael Fouré, Franck Blindauer, Céline Giraudeau, Hussein El-Saghire, Mourad Bouhadjar, Cynthia Calligaro, Tania Sorg, Philippe Choquet, Thierry Vandamme, Christophe Ferrand, Jacques Marescaux, Thomas F. Baumert, Michele Diana, Patrick Pessaux, Eric Robinet

**Affiliations:** 1 INSERM, U 1110, 67000 Strasbourg, France; 2 University of Strasbourg, 67000 Strasbourg, France; 3 Department of Hepatobiliary and Pancreatic Surgery, Second Affiliated Hospital of Kunming Medical University, Kunming, 650500, Yunnan, People’s Republic of China; 4 IHU-Strasbourg, Institute of Image-Guided Surgery, 67000 Strasbourg, France; 5 Pathology Department, University Hospital of Strasbourg, 67000 Strasbourg, France; 6 Mouse Clinical Institute, 67400 Illkirch, France; 7 Pôle Hépatodigestif, Unité Hépatologie, Hôpitaux Universitaires de Strasbourg, 67000 Strasbourg, France; 8 Research Institute against Cancer of the Digestive System (IRCAD), 67000 Strasbourg, France; 9 Functional Unit 6237, Preclinical Imaging, Hôpitaux Universitaires de Strasbourg, 67000 Strasbourg, France; 10 National Center for Scientific Research (CNRS), ICube, MMB team, 67000 Strasbourg, France; 11 Medical Faculty, Fédération de Médecine Translationnelle de Strasbourg (FMTS), 67000 Strasbourg, France; 12 National Center for Scientific Research (CNRS), UMR 7199, 67400 Illkirch, France; 13 LLTech SAS, 75014 Paris, France; 14 French Blood Agency Bourgogne/Franche-Comté, 25000 Besançon, France; 15 INSERM, U 1098, 25000 Besançon, France; 16 Université de Franche-Comté, 25000 Besançon, France

## Abstract

The development of multimodal strategies for the treatment of hepatocellular carcinoma requires tractable animal models allowing for advanced *in vivo* imaging. Here, we characterize an orthotopic hepatocellular carcinoma model based on the injection of luciferase-expressing human hepatoma Huh-7 (Huh-7-Luc) cells in immunodeficient mice. Luciferase allows for an easy repeated monitoring of tumor growth by *in vivo* bioluminescence. The intrahepatic injection was more efficient than intrasplenic or intraportal injection in terms of survival, rate of orthotopic engraftment, and easiness. A positive correlation between luciferase activity and tumor size, evaluated by Magnetic Resonance Imaging, allowed to define the endpoint value for animal experimentation with this model. Response to standard of care, sorafenib or doxorubicin, were similar to those previously reported in the literature, with however a strong toxicity of doxorubicin. Tumor vascularization was visible by histology seven days after Huh-7-Luc transplantation and robustly developed at day 14 and day 21. The model was used to explore different imaging modalities, including microtomography, probe-based confocal laser endomicroscopy, full-field optical coherence tomography, and ultrasound imaging. Tumor engraftment was similar after echo-guided intrahepatic injection as after laparotomy. Collectively, this orthotopic hepatocellular carcinoma model enables the *in vivo* evaluation of chemotherapeutic and surgical approaches using multimodal imaging.

Hepatocellular carcinoma (HCC) is the second most common cause of cancer-related death^1^ and the leading cause of death among cirrhotic patients^2^. Despite an increasing therapeutic armamentarium, global survival rates remain poor. HCC management requires a multidisciplinary approach, involving surgeons, oncologists, interventional radiologists, for the complex diagnostic and therapeutic algorithms related to tumor size, location, liver function, performance scores, and to criteria for Orthotopic Liver Transplantation (OLT). Surgery is the most effective treatment, particularly in case of localized tumors and when liver function is not a limiting factor for extensive hepatectomies. Locoregional interventional therapies (Cryoablation, Percutaneous Ethanol Injection, Trans-arterial Chemoembolization and Radio-Frequency Ablation, High Intensity Focused Ultrasound), are used as complementary treatments in case of multilobular or advanced disease, in sequential or combined protocols^3^. With regards to chemotherapy, the multi-kinase inhibitor Sorafenib obtained survival improvements, however limited by adverse effects, in patients presenting locally advanced or metastatic HCC^4^.

The quest for an optimized, well-tolerated, minimally invasive, cost-effective therapy for HCC management, remains an unmet clinical need and active research topic. Relevant preclinical animal models are crucial for the development of innovative approaches to HCC, including irreversible electroporation, gene therapy, immunotherapy, and other theranostics. So far, many murine models have been used to define the pathogenesis of HCC and have contributed to the current knowledge of HCC. Those models are based on chemical induction, genetic modification (including transgenic expression of host oncogenes, viral genes or growth factors) or xenogeneic tumor cell transplantation^5^. The ideal HCC animal model should gather several characteristics:

It should reproduce the etiologies of HCC, such as Hepatitis B or C Virus-induced HCC;

It should recapitulate all steps of HCC progression and pathogenesis, including fibrosis and cirrhosis;

It should be humanized, optimally at the cellular level, or at least at the genetic level;

It should be sized to enable the assessment of therapeutic options requiring human-sized instruments, such as interventional radiology approaches;

It should be standardized, reproducible on a large scale, and affordable.

Such a model accumulating all these features is not available, and is difficult to obtain. For this reason, different complementary models are used to evaluate fundamental parameters (e.g. biodistribution or pharmacokinetics/pharmacodynamics) for drug development or efficacy and for the safety profile of innovative interventional therapeutic and diagnostic modalities. Additionally, in the current era of precision medicine, image guidance plays a crucial role in tailoring patient-specific approaches and consequently, a relevant HCC model is also required to improve the accuracy of imaging and to design new contrast agents or delivery systems. The aim of the present study was to characterize a xenogeneic orthotopic model of HCC through an intrahepatic injection of hepatoma Huh-7 cell line^6^ expressing the luciferase reporter gene, on the basis of various preclinical and clinically relevant imaging systems.

## Results

### Comparison of engraftment levels according to the injection site

#### Intrasplenic injection

We first characterized the model as a subcutaneous tumor model (Supplementary Data & Supplementary Fig. 1). Then, as a first model of orthotopic humanized HCC, Huh-7-Luc cells were transplanted in SCID-bg mice by means of intrasplenic injections^7^. In a series of 90 SCID-bg mice, the mortality rate was 10%, and the overall rate of tumor engraftment (i.e., the percentage of mice with detectable tumors, whatever their location (inside or outside of the liver or both)), was 68% of surviving mice. However, it was estimated, from bioluminescence images, that a tumor engraftment localized exclusively in the liver was obtained in 30% of surviving mice only, which is congruent with data available in the literature^8^. In order to limit the interference of the white-colored fur on *in vivo* bioluminescence and fluorescence assays, we decided to shift to the NMRI-nu mouse strain. In a smaller series of NMRI-nu mice (n = 17), there was no mortality and the overall rate of tumor engraftment was 88%, with 41% of mice having a tumor engraftment localized exclusively in the liver.

Overall, intrasplenic injection was associated with ≤10% intraoperative mortality (i.e. within 48 hours post-transplantation) and a high efficacy of Huh-7-Luc engraftment (Table 1). However, tumor cells engrafted not only in the liver, but also outside of the liver, mostly in the spleen and/or in the peritoneum. In order to determine whether the low engraftment rate resulted from a problem of engraftment of transplanted cells or if the cells get stuck in the spleen, 16 mice were transplanted with Huh-7-Luc cells. At different time points after intrasplenic injection (3 hours, day 1, day 3, and day 7), four mice were injected with luciferin and the livers and spleens were harvested for *ex vivo* detection of their luciferase activity. The site of luciferase activity was cut into two parts for human pan-cytokeratin immunostaining and human cell quantification by qPCR. When the luciferase activity was not detectable in the liver, the site of injection (identified by necrotic areas) was used instead. Huh-7-Luc cells were observed in all spleen samples, from 3 hours to day 7 (Supplementary Fig. 2a, lower right panels) and were mostly located in the red pulp. In contrast, few Huh-7-Luc cells were observed occasionally in the portal veins of some livers, suggesting that they were stuck in the spleen, while a strong immunostaining of murine biliary ducts and a weaker immunostaining of some bile duct-proximal murine hepatocytes were observed in the livers, due to a cross-reaction of the antibody (Supplementary Fig. 2a, lower left panels). Monitoring of Huh-7-Luc cells by qPCR in explanted livers (Supplementary Fig. 2b) and spleens (Supplementary Fig. 2c) showed that Huh-7-Luc cells were detectable in both organs, with a trend towards a decreased median frequency of human cells in the liver at day 1 and day 3, while the median frequency of human cells remained stable in the spleen. Altogether, these results suggest that most cells get stuck in the spleen and that part of the cells present in the liver die after injection.

#### Intraportal injection

In a first attempt, intrasplenic injections were replaced by intraportal injections in order to improve intrahepatic engraftment (Supplementary Fig. 3). This procedure was associated with an engraftment rate which is similar to intrasplenic injection (Table 1). However, Magnetic Resonance Imaging (MRI) monitoring showed that most tumors had a periportal location rather than an intrahepatic location (Supplementary Fig. 4). In addition, intraportal injection was also associated with an increased mortality rate, leading to a significantly lower engraftment yield (Table 1) and it was a more complex method. As a result, it was not further considered.

#### Intrahepatic injection

We then tested intrahepatic injections in the left lobe after laparotomy (Supplementary Fig. 5). The tumor engraftment rate, evaluated by BLI (86.0%), was significantly higher than after intraportal injection (69.3%; p = 0.0009, chi-square test) or intrasplenic injection (71.4%; p = 0.0025, chi-square test), with a lower intraoperative death rate than after intraportal injection (5.2% vs. 42.9%, respectively; Table 1). After an initial loss of weight of less than 10% 24 hours after surgery, the mice recovered their initial weight at day 3 (Supplementary Fig. 6). Consequently, only intrahepatic injections were further considered. In the following sections, intrahepatically injected NMRI-nu mice were evaluated with different imaging modalities, alone or in combination, as indicated in Table 2.

### Kinetic of tumor growth

**Figure 1:**
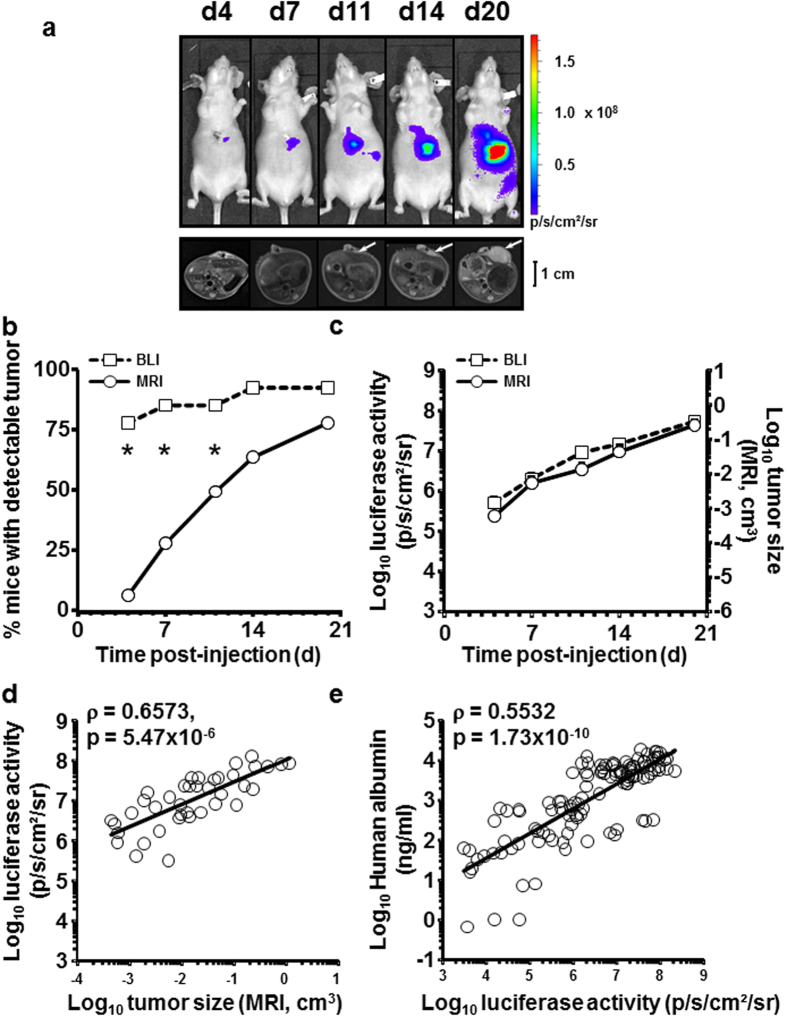
Correlation between the tumor size, evaluated by MRI, and luciferase activity, evaluated by BLI. (**a**) Monitoring of a representative animal, evaluated at the indicated time points by BLI (upper panels, coronal plane) and MRI (lower panels, axial plane). Bar size: 1 cm. (**b**) BLI (squares, dashed line) is more sensitive than MRI (circles, full line) to detect tumors, as evaluated by the frequency of mice with detectable tumors (n = 14 mice evaluated by BLI and MRI). *P < 0.05 (chi-square test) (**c**) The increase in tumor size, evaluated by MRI (circles, full line), is superimposable with the evolution of the tumor’s luciferase activity, evaluated by BLI (squares, dashed line), among mice with tumors simultaneously detected by MRI and BLI. Data are expressed as mean±SE of BLI (p/s/cm^2^/sr) or MRI (size in cm^3^) in detectable mice (BLI: n = 11, 12, 12, 13, 13 and MRI: n = 1, 5, 9, 11, 13 detectable mice at days 4, 7, 11, 14, 20 respectively). (**d**) Positive correlation between tumor size, evaluated by MRI (cm^3^), and luciferase activity, evaluated by BLI (p/s/cm^2^/sr). Pearson correlation test: linear correlation coefficient: ρ = 0.6573, p = 5.47 × 10^−6^. Data in A-D are from 13 mice transplanted by intrahepatic injection of Huh-7-Luc cells and monitored in parallel by BLI and MRI. (**e**) Positive correlation between luciferase activity and serum levels of human albumin. Pearson correlation test: linear correlation coefficient: ρ = 0.5532, p = 1.73 × 10^−10^, n = 114 determinations from 82 mice used in 7 independent experiments the serum of which was harvested (at different time points after intrahepatic transplantation of Huh-7-Luc cells) for human albumin quantification at time of BLI analysis.

In order to characterize the kinetic of tumor growth, 14 mice were injected intrahepatically with Huh-7-Luc cells. Tumor engraftment was obtained in 13/14 mice, with intrahepatic tumor in all 13 mice and up to 5 nodules in the liver (Supplementary Fig. 7). Additional extrahepatic tumors were observed in 5/13 mice. Tumor growth was monitored in parallel by means of BLI and MRI from day 4 to day 20 post-injection, as shown in Fig. 1a for one representative mouse. Due to logistical constraints, MRI was performed in this experiment with a clinical grade system and without injection of contrast agent. *In vivo*, BLI was more sensitive than MRI, since tumor cells were detectable significantly more frequently by BLI than by MRI (Fig. 1b) but, in tumor-detectable mice, the kinetics of tumor growth evaluated by MRI and BLI were superimposable (Fig. 1c). Additionally, a significant positive correlation (r = 0.6573, p = 5.47 × 10^−6^, Pearson correlation test) was observed between luciferase activity, quantified by BLI and tumor size, quantified by MRI (Fig. 1d). The volume of the tumors was extracted from manual segmentations performed directly on MRI slices. This allowed to establish an approximate correspondence between luciferase activity and tumor size (Table 3). It prompted us to propose to our local ethics committee the luciferase activity value of 10^8^ p/s/cm^2^/sr as the endpoint value for animal experimentation with this model. We also evaluated the possibility to use the human albumin, produced by Huh-7 cells, as a serum marker of tumor growth. Sixty serum samples were harvested from 31 mice at different time points after Huh-7-Luc cell transplantation, in parallel to BLI acquisition. As shown in Fig. 1e, there was a significant positive correlation between luciferase activity and serum levels of human albumin (r = 0.3704, p = 3.58 × 10^−3^, Pearson correlation test).

### Histological analysis

**Figure 2:**
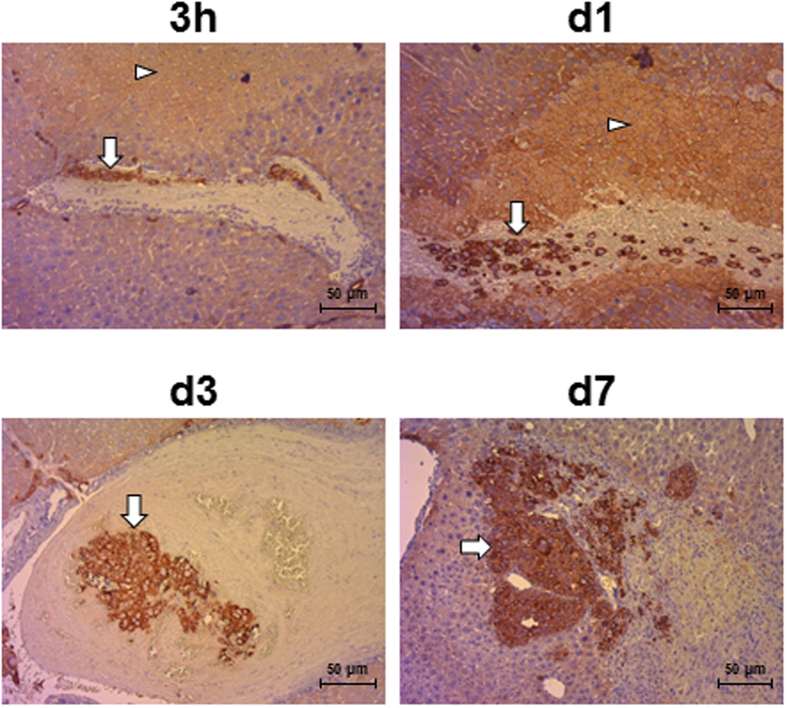
Location of Huh-7-Luc cells after intrahepatic injection. At the indicated time after intrahepatic injection, the liver was harvested to perform a pan-human cytokeratin immunostaining. Huh-7- White arrowheads: necrotic areas in hepatic parenchyma; white arrows: tumor cells, identified by pan-human cytokeratin immunostaining. Huh-7-Luc cells are visible as clots in portal veins until day 3 and as tumors at day 7. Images are from one representative mouse out of four mice per time point. Scale bars: 50 μm.

Tumor vascularization is a key parameter for the study of innovative HCC treatments, such as immunotherapy cell products^7^ or nanovectors. In order to characterize the kinetic of tumor neovascularization, 16 mice were injected, in a first experiment, with Huh-7-Luc cells and monitored by BLI at days 1, 2, 3, 4, 7, 11, 14, and 21 (Supplementary Fig. 8a). At each time point, the two mice exhibiting the highest and lowest BLI value were sacrificed for histological analysis. In a second experiment, 16 mice were injected with Huh-7-Luc cells and four mice were analyzed by BLI (Supplementary Fig. 8b) and sacrificed 3 hours after injection and at day 1, day 3, and day 7 for histological analysis. A necrosis was observed in the hepatic parenchyma as early as 3 hours after injection (Fig. 2) and reached a maximum level at day 4 and day 7 (Supplementary Fig. 9, white arrowheads). The necrotic parenchyma was surrounded by an inflammatory infiltrate (Supplementary Fig. 9, day 1 and day 4, lower panels). This necrosis resulted from Huh-7-Luc cell-induced embolism of portal veins (Fig. 2 and Supplementary Fig. 9, day 2, lower panel), as it was not observed upon intrahepatic injection of saline alone (data not shown). Huh-7-Luc cells were occasionally observed in the liver parenchyma at early time points (Supplementary Fig. 9d1) but were mostly observed as clots in portal veins (Fig. 2). In the two experiments, BLI monitoring at early time points (i.e., during the first week post-injection) showed an initial decrease in luciferase activity, followed by BLI recovery (Supplementary Fig. 8a,b). In order to determine whether this decrease in luciferase activity resulted from an insufficient oxygen supply, due to necrosis, or from Huh-7-Luc cell death, a human-specific qPCR was performed in order to quantify the frequency of human cells in the liver. Huh-7-Luc cells were detected at frequencies ranging from 2.2% to 8.6% 3 hours after injection. However, they were detected at lower frequencies, ranging from non-detectable to 0.7%, at day 1, day 3, and day 7 (Supplementary Fig. 8c). This result suggests that most Huh-7-Luc cells die upon injection and that only a minority of cells can seed and grow after one week.

**Figure 3:**
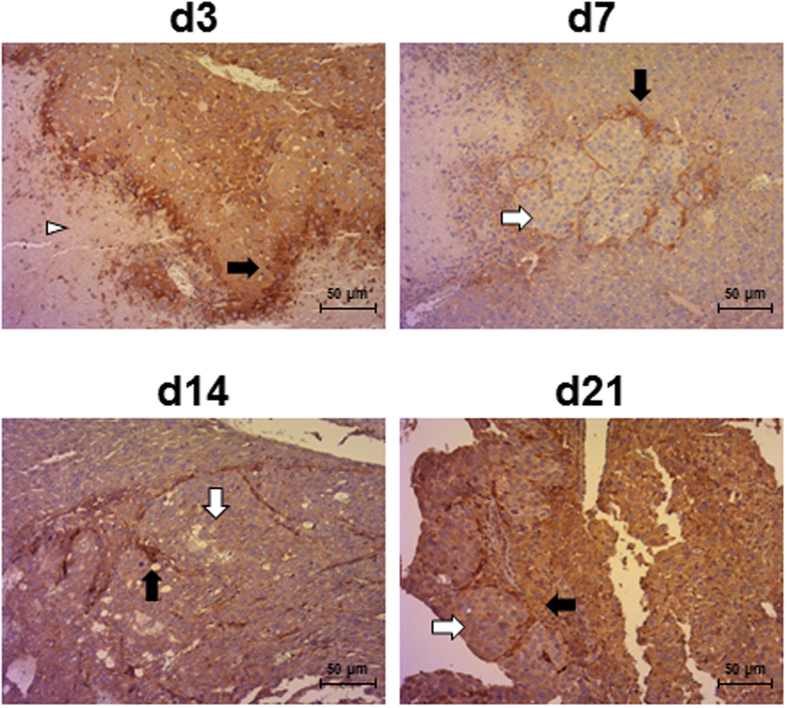
VEGF immunostaining of Huh-7-Luc tumors. Human VEGF-A immunostaining of the samples shown in Supplementary Figures 4 and 5 was performed at the indicated time points. White arrowheads: necrotic areas in hepatic parenchyma; white arrows: tumor cells; black arrows: VEGF-A staining. Scale bars: 50 μm.

Indeed, tumor nodules were observed in the parenchyma from day 7 (Fig. 2,3 and Supplementary Fig. 9) and were surrounded by neovessels, as demonstrated by immunostaining of VEGF-A, an angiogenic factor (Fig. 3 and Supplementary Fig. 10). Before day 7, a VEGF-A staining was observed only on hepatocytes surrounding necrotic areas (Fig. 3 and Supplementary Fig. 10). At days 14 and 21, tumors were highly vascularized (Supplementary Figs 9 and 10) but also exhibited large necrotic areas. Upon progressive elimination of necrotic cells, these areas left place for hemorrhagic lakes at day 21 (Supplementary Figs 9 and 10). *In vivo* probe-based Confocal Laser Endomicroscopy (pCLE) performed on livers at day 14 after Huh-7-Luc injection also allowed to observe the disruption of normal regular plates of hepatocytes in liver tumors. Additionally, a dense, disorganized neovascularization was clearly visible at the surface of the tumors (Supplementary Video 1), while the sinusoids converging through the centrilobular vein in the healthy liver parenchyma were thin and regular (Supplementary Video 2).

### Comparison of Light-Coherence Tomography scan imaging with histology

**Figure 4:**
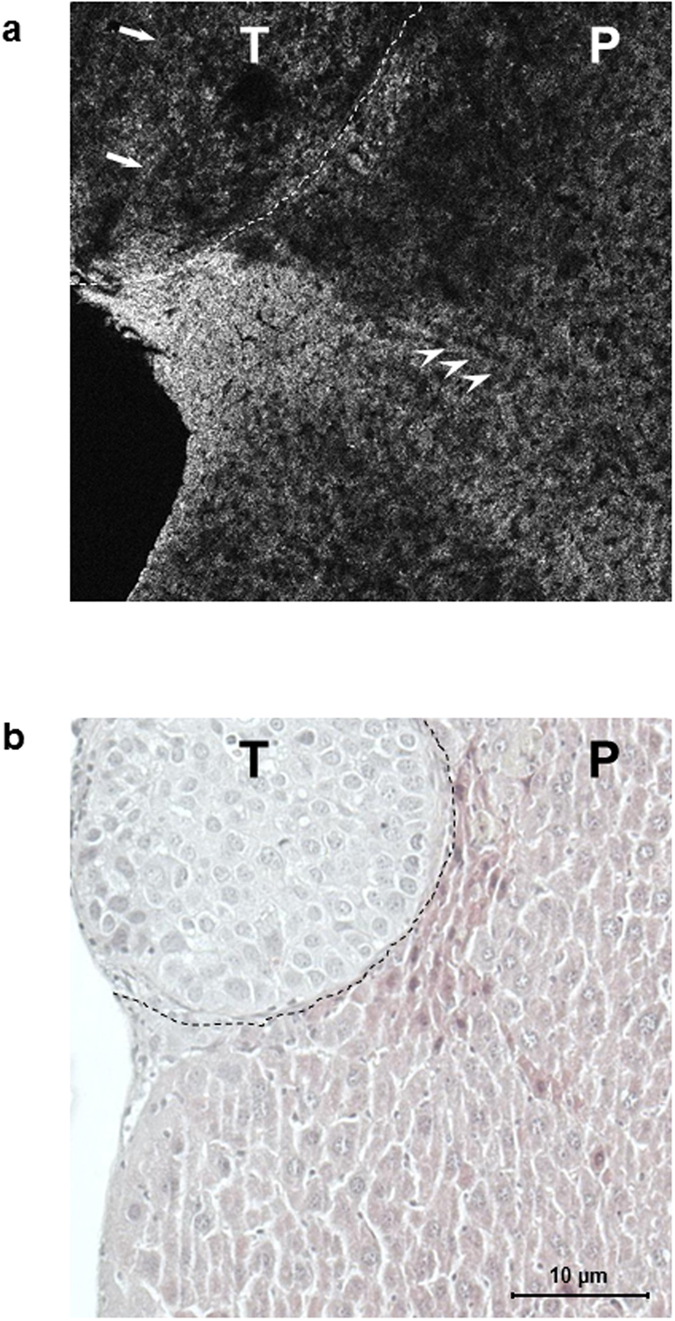
Light-CT scan of Huh-7-Luc tumor. A liver harvested 14 days after Huh-7-Luc injection was evaluated by Light-CT scan imaging, then fixed and analyzed by histology. The same area analyzed by Light-CT scan (upper panel) is shown after H/E staining (lower panel). Normal hepatocytes (white arrowheads: nuclei) appear regularly distributed while tumor cells (white arrows) are more heterogeneous. The hypereosinophilic area (black dotted line) observed after H/E staining between tumor (T) and normal parenchyma (P) appears hyperdense under Light-CT scan (white dotted line). Bar size: 10 μm.

Light-Coherence Tomography (Light-CT) scan imaging allows for volumetric image capture on fresh tissue samples, without the requirement of tissue fixation, allowing for real-time histological analysis. Within a few minutes, the tissue architecture and microstructure is revealed, in 2D and 3D, without damaging or modifying the sample under analysis. To evaluate this innovative imaging method, 2D (Fig. 4a & Supplementary Video 3) and 3D (Supplementary Video 4) Light-CT scan imaging of livers harvested at day 14 after Huh-7-Luc cell injection was performed after division of the liver through the middle of the tumor, in order to image both healthy and tumoral areas. The samples were then fixed and submitted to histological analysis after H/E staining (Fig. 4b). In the healthy area, normal eosinophilic hepatocytes appear regularly distributed after H/E staining and the corresponding area of Light-CT scan imaging shows a regular distribution of hepatocytes nuclei (Fig. 4), while the tumor appears more heterogeneous. Although further analyses are required to evaluate the interest of Light-CT scan imaging, these preliminary results suggest that a good correspondence could be achieved between this method and standard histology performed by H/E staining.

### Evaluation of a contrast agent for X-ray microtomography

**Figure 5:**
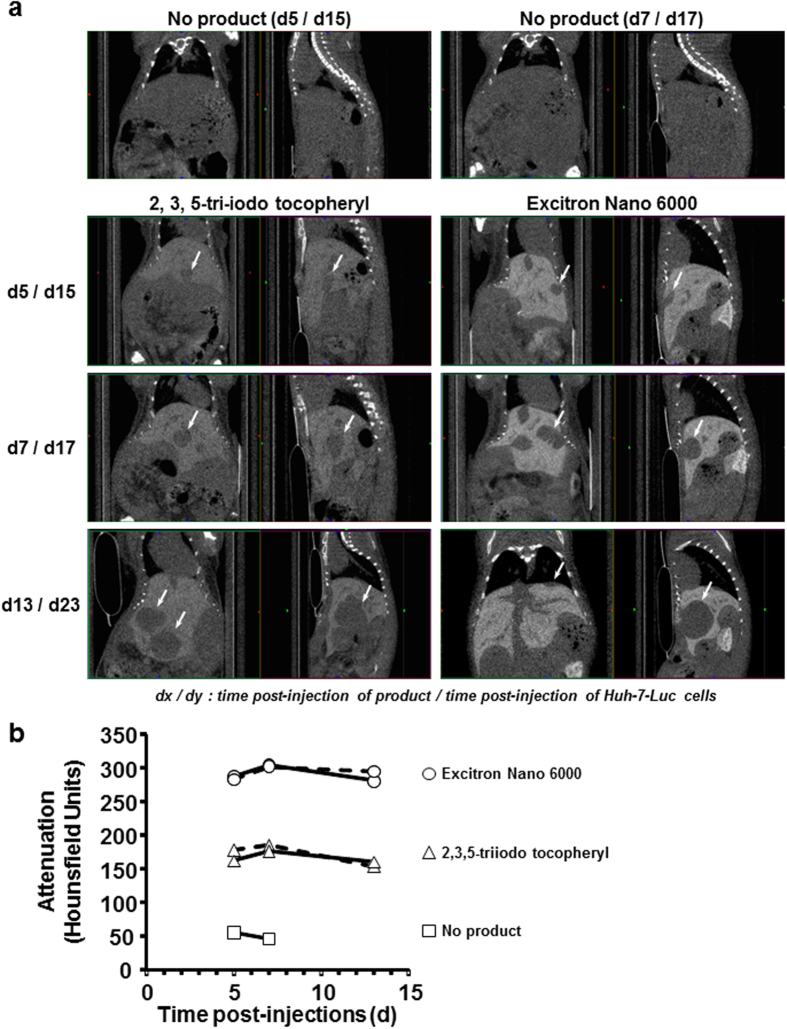
Comparison of two contrast agents by μCT-scan. Ten days after intrahepatic injection of Huh-7-Luc cells to 5 mice, 4 μl/g body weight of α-tocopheryl 2, 3, 5-triiodobenzoate (n = 2), Excitron Nano 6000 (n = 2) were injected into the lateral tail vein and no injection was performed in a control mouse. μCT-scanning was performed at days 5, 7, and 13 after contrast agent injection, i.e. after 15, 17 and 23 days after Huh-7-Luc injection. (**a**) μCT-scan imaging of one control mouse (upper panels) and one mouse injected with α-tocopheryl 2, 3, 5-triiodobenzoate (left panels) or Excitron Nano 6000 (right panels). Each panel shows the coronal (left image) and sagittal (right image) plane. White arrows indicate the tumors. (**b**) Quantification of the liver contrast in the hepatic parenchyma of mice injected with α-tocopheryl 2, 3, 5-triiodobenzoate (triangles, n = 2), Excitron Nano 6000 (circles, n = 2) or saline (squares, n = 1).

**Figure 6:**
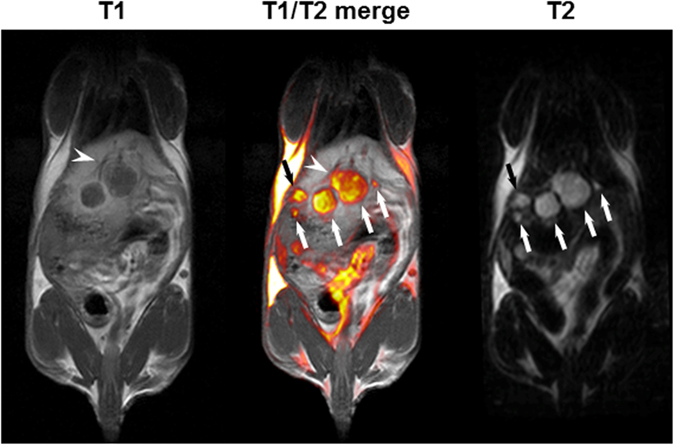
Merge of T1/T2 MRI sequences. Magnetic resonance imaging of a mouse (same animal as in Fig. 5a, left panel) in the coronal plane, was performed with a preclinical MRI system. Five tumor nodules (arrows) are visible on the T2 sequence (right), but the smallest nodules are not or barely visible on the T1 sequence (left). Conversely, the related tumor vascular supply (arrowhead) is more easily detectable on the T1 sequence than on the T2 sequence. Merging of T1 and T2 sequences (middle) allows for the simultaneous and enhanced visualization of tumor nodules and the related tumor vascular supply.

As an example for the use of our model, we blindly compared the α-tocopheryl 2, 3, 5-triiodobenzoate, a potentially new contrast agent for X-ray microtomography (μCT) scan imaging^9^, with Excitron Nano 6000, used as a positive control product. Five mice with 10-day Huh-7-Luc intrahepatic tumors were injected with either α-tocopheryl 2, 3, 5-triiodobenzoate (n = 2) or Excitron Nano 6000 (n = 2) or were not injected (n = 1). A persistent enhancement of the hepatic parenchyma contrast was observed with both products (Fig. 5a), although to a higher intensity with Excitron Nano 6000 (Fig. 5b). Indeed, a calibration of each product demonstrated that Excitron Nano 6000 and α-tocopheryl 2, 3, 5-triiodobenzoate contained 171 and 103 mg/mL of Iodine-equivalent respectively (Supplementary Fig. 11). Consequently, 684 and 431 mg/kg of iodine-equivalent were administered to the mice, leading to a stronger signal with Excitron nano 6000 than with α-tocopheryl 2, 3, 5-triiodobenzoate. Given the volumes of injections used, we did not consider to increase the dose of α-tocopheryl 2, 3, 5-triiodobenzoate in further experiments, for ethical reasons. However, tumors were visible in negative contrast (Fig. 5a) to a sufficient extent to allow for performing 3D reconstruction (Supplementary Video 5). These mice were also imaged with a preclinical MRI system after injection of MultiHance^®^ contrast agent, allowing to perform 3D reconstruction (Supplementary Videos 6 and 7) and T1/T2 fusion imaging (Fig. 6), enabling the simultaneous and enhanced visualization of tumor nodules and the related tumor vascular supply.

### Ultrasound image-guided intrahepatic cell injection

**Figure 7:**
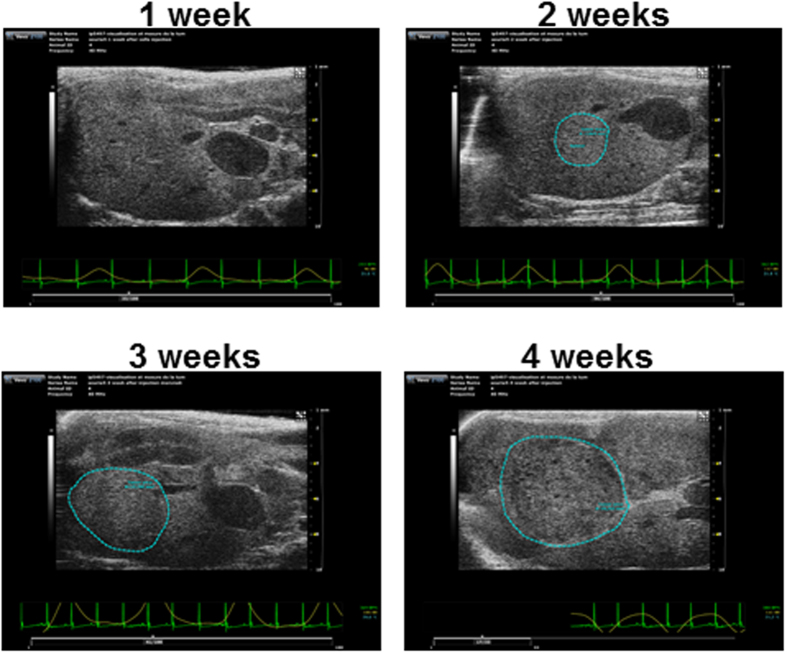
Echo-guided intrahepatic injection of Huh-7-Luc cells. US imaging monitoring of tumors after echo-guided injection of Huh-7-Luc cells. The tumor is surrounded in blue. Tumor area is 7.1, 19.8, and 35.3] cm^2^ at week 2, 3, and 4 respectively.

Intrahepatic injection of Huh-7-Luc cells was performed after laparotomy, which is a relatively invasive procedure. Consequently, we evaluated the possibility to perform a less invasive intrahepatic injection under ultrasound (US) image guidance (Supplementary Fig. 12 and Supplementary Video 8). In 3 independent experiments, a total of 18 mice were injected under echography with a Vevo Imaging system and using an injection mount device. Tumor growth monitoring by echography (and confirmation at necropsy) revealed an intrahepatic tumor growth in 16 mice (Fig. 7 and Supplementary Video 9), an extrahepatic growth in one mouse and a lack of engraftment in the last one.

### Response to treatment and evaluation of additional cell lines

Additional experiments, reported as Supplementary Data, include the response of Huh-7-Luc tumors to standard of care treatments, sorafenib and doxorubicin (Supplementary Figure 13) and evaluation of the engraftment of additional cell lines (Supplementary Figure 14).

## Discussion

Numerous HCC tumor models in mice were described in the literature (reviewed for HCC models in reference^5^), including xenotransplantation of human tumor cells^10^,^11^. These human xenograft models can be divided into Patient-Derived Xenograft (PDX) and Cell-Derived Xenograft (CDX) models. Patient-Derived Xenograft models, based on transplantation into immunocompetent recipient mice of primary tumors harvested directly from the patient, are considered more pertinent than CDX models, based on transplantation of established cell lines and cultured *in vitro* (such as Huh-7 cells)^12^,^13^. Indeed, PDX models recapitulate the genetic heterogeneity of genomic mutations observed in a tumor more precisely and subsequently better reflect, or even predict, drug response than CDX models^13^,^14^. However, some limitations, including high cost, long latency periods for tumor growth, variable engraftment rate after transplantation and more specialized skills, have limited the use of PDX models. As a result, despite being less relevant, CDX models are still largely used as they can remain useful and informative as a first step for the evaluation of a new therapeutic approach. Both PDX and CDX models can be used as orthotopic models, i.e. located in their original organ location, or as subcutaneous models. The former is usually considered more relevant since it reflects tumor microenvironment interactions more precisely. However, the latter is easier to transplant and to monitor.

Consequently, although considering the development and use of PDX models for our research projects, we decided to use CDX models of HCC in parallel with PDX models, as a first methodological step for proof-of-concept studies^7^,^15^. Since Huh-7 cells are commonly used for *in vitro* and *in vivo* studies^16^,^17^,^18^,^19^,^20^,^21^,^22^, we used this cell line as a target for gene transfer of the luciferase reporter gene, allowing for a non-invasive monitoring of tumor growth by means of BLI. This approach is indeed in agreement with the ‘’3R rules” of animal experimentation (Refine, Reduce, Replace) since, as a non-invasive method, BLI allows to refine the monitoring of tumor growth (as compared to biological monitoring, such as serial blood harvesting for quantification of serum markers: human serum albumin, alpha-fetoprotein, etc.) and to reduce the number of animals required per group, as the same mouse can be monitored several times (as compared to gross examination of tumor volume or weight after liver explant). Alternative non-invasive imaging methods, such as MRI, μCT-scan imaging or US imaging, may be used adequately for monitoring but also for cell injection. In this respect, echo-guided injection of Huh-7-Luc cells, by avoiding the performance of a laparotomy, is less painful and further refines the procedure of intrahepatic transplantation. We recently reported a μCT-scan image-guided robotized injection device which may also be used to this aim^23^.

When establishing a luciferase-based tumor model, a careful evaluation of the model should be made before using it routinely. As an example, we generated two luciferase-expressing Huh-7 cell lines, one after retroviral-mediated transduction (Huh-7-Luc cells) and a second one after lentiviral-mediated transduction (Huh-7-lenti-Luc cells). Although Huh-7-lenti-Luc cells presented approximately 2-logs higher luciferase activity than Huh-7-Luc cells on a per-cell basis, their luciferase activity remained stable after orthotopic transplantation and no increase in tumor size was observed. This absence of correlation between luciferase activity and tumor size suggested that the transplanted cells survived but did not grow after intrahepatic injection (Supplementary Fig. 13). Conversely, a constant increase in tumor size was observed (as shown by MRI - Fig. 1a - or μCT-scan imaging - Fig. 5a) after Huh-7-Luc cell intrahepatic injection and a positive correlation between luciferase activity and tumor size demonstrated the usefulness of this model. The discrepancy between Huh-7-Luc and Huh-7-lenti-Luc cells may be due to the selection, after transduction, of clones with different growth abilities. Similarly, a low efficacy of tumor growth was observed with additional cell lines (Supplementary Fig. 13), especially with HepG2 cells, a hepatoma cell line the *in vivo* tumorigenicity of which is controversial^16^,^24^,^25^,^26^,^27^. This might be due to genetic drift of the cell line from a laboratory to another or to insufficient number of injected cells. Indeed, high number of HepG2 cells need to be injected in order to develop a tumor^25^,^27^. Thus, an improvement in engraftment might be reached for these cell lines by modifying some parameters, such as the number of transplanted cells or use of Matrigel. This clearly demonstrates that each model must be validated before use. The kinetic of Huh-7-Luc tumor growth, evaluated through its luciferase activity (Fig. 1c), is similar to the one reported by other groups^20^. From an ethical standpoint, luciferase activity values might be used as an anticipated experiment endpoint. However, the values we have reported (Table 3) should be considered as merely indicative since a high heterogeneity exists among CDX models in terms of luciferase expression and tumor growth.

Huh-7 cells have been used *in vivo* as surrogate models for HVC or HBV infection, two main etiologies of HCC, which could be beneficial over PDX models. However, our model is limited by the lack of genetic heterogeneity, as transduced Huh-7 cells were cloned before cryopreserving a master cell bank. Other limitations include the absence of immune system and the fact that the tumor develops on a healthy liver, a clinical setting rarely observed. A recent study reported an orthotopic HCC model on fibrotic liver in immunocompetent recipients by intrahepatic injection of a syngeneic murine HCC line in CCl4-pretreated mice. CCl4 is a potent hepatotoxic drug inducing fibrosis, cirrhosis, and ultimately HCC. Due to its high toxicity, its use is no longer possible in Europe for safety reasons. In order to similarly establish a human CDX or PDX models on fibrotic livers, we wish to evaluate, as an alternative, the possibility to treat mice with an intraperitoneal injection of diethylnitrosamine as fibrotic inducer, several weeks before intrahepatic Huh-7-Luc cell injection. Establishing an immune competent CDX or PDX model by human CD34+ hematopoietic stem cell transplantation in highly immunodeficient NSG or NOG mice recipients has already been achieved. However, it is highly challenging, especially if one wish to further include a fibrosis. Consequently, different levels of complexity may be desired or achieved according to the question to be raised, including induction of fibrosis, humanization of the tumor with a CDX or PDX model, humanization of the liver compartment with primary human hepatocytes (in order to further support HBV or HCV infection) or humanization of the immune system (in order to support immune responses to the tumor or viral infections). As a result, the Huh-7-Luc model presented in our study is an easy-to-set-up model which may be used as a platform to further complexify the model, for use as surrogate viral infection or HCC model in fibrotic liver.

## Methods

### Cell lines

Huh-7 cells (Japanese Collection of Research Bioresources Cell Bank, Osaka, Japan) stably expressing luciferase (Huh-7-Luc) were produced by transduction using a retroviral vector generated from a luciferase-encoding pCLNCX vector (Dr. Lorang, NIH, Bethesda, MD, USA) and cloning by limiting dilution. The clone with the highest luciferase activity was amplified to produce a Master Cell Bank and secondary Working Cell Banks and was characterized as described in Supplementary Data and Supplementary Fig. 15. Additional cell lines used were SK-Hep1 transduced with the same pCLNCX vector (SK-Hep1-Luc cells), commercially available luciferase-expressing HepG2 cells (HepG2-Luc cells; Perkin Elmer, Waltham, MA) and Huh-7, PLC/PRF/5 and HepG2 cells (American Type Cell Culture, LGC Standards, Molsheim, France) transduced with a luciferase-encoding lentiviral vector using a HIV-gag-pol (A. Meyerhans, Department of Experimental and Health Sciences, Universitat Pompeu Fabra, Barcelona, Spain), pMD2G VSV-G-Env (D. Trono, Laboratory of Virology and Genetics, Ecole Polytechnique Fédérale de Lausanne, Switzerland) and pLenti CMV V5-LUC (Addgene, Cambridge, MA) plasmids, as previously described^28^ (Huh-7-lenti-Luc, PLC/PRF/5-lenti-Luc, and HepG2-lenti-Luc cells). All cell lines were cultured in Dulbecco’s Modified Eagle Medium (DMEM, PAA Laboratories) supplemented with non-essential amino acids (GIBCO^®^, Invitrogen, Cergy Pontoise, France), 10 μg/mL gentamycin (GIBCO^®^) and 10% fetal bovine serum (PAN Biotech GmbH, Aidenbach, Germany).

### Animal experimentation

Animal experimentations were performed in accordance with European recommendations (Directive 2010/63/UE, September 22, 2010) and French regulations (Décret 2013–118, February 1, 2013) and received the approval of the local ethics committee (Comité Régional d’Ethique en Matière d’Expérimentation Animale de Strasbourg, approvals No. AL/33/40/02/13, AL/34/41/02/13, 00465.02, and 03111). Six- to twelve-week-old SCID-bg (C.B-Igh-1b/GbmsTac-Prkdcscid-Lystbg N7) male and female mice purchased from Taconic (Hudson, NY) and bred in our facility and 6- to 12-week-old NMRI-nu (Rj:NMRI-Foxn1nu/Foxn1nu) female mice purchased from Janvier Labs (Le Genest Saint Isle, France) were used for experimentation. All surgical procedures were performed under 1 to 3% isoflurane anesthesia (Axience Laboratories, Pantin, France) with a 2 to 3 L/min air flow rate, with or without a 0.2 L/min O_2_ flow rate. Analgesia was performed at the start of the procedures, directly in the abdominal cavity, by intraperitoneal or local administration of buprenorphine (Buprecare^®^, Axience Laboratories) at a dose of 0.1 mg/kg. Intraperitoneal injections of buprenorphine at the same dose were performed eight hours later and, if required, the following days. Paracetamol (Doliprane, Sanofi-Aventis, Paris, France) was given at a dose of 1 mg/mL in drinking water until the end of the experiment. For ethical reasons, mice were not monitored until tumor-related death but were euthanized at the end of experiments or when endpoints were reached.

### Huh-7-Luc cell transplantation

Huh-7-Luc cells were either orthotopically transplanted by intrasplenic injection, intraportal injection, intrahepatic injection after laparotomy or echo-guided intrahepatic injection or were injected subcutaneously. For all injection procedures, the skin of the mice was sterilized with Betadine and ethanol before and after the surgical procedure and 1 × 10^6^ Huh-7-Luc cells resuspended in a 50 μL culture medium were injected. At the end of each manipulation, mice were placed on a heat pad and injected subcutaneously with a Buprenorphine analgesic (0.05 mg/kg) before awakening and becoming mobile. They were then returned to their regular cage once awakened. All mice received paracetamol (Doliprane, Sanofi-Aventis, Paris, France) 1 mg/mL *ad libitum* in drinking water until the end of the experiments. All procedures were performed at the animal experimentation platform of the National Institute of Health and Medical Research, Institute of Viral and Liver Disease (UMR 1110), except for echo-guided injections, performed at the imaging platform of the Mouse Clinical institute (MCI). Detailed procedures of cell injection are described as Supplementary Data.

### *In vivo* imaging

The following imaging modalities were performed using either clinical or preclinical imaging systems at several imaging platforms located at or in the vicinity of Strasbourg, France:

large animal imaging platform of the Institute of Image-Guided Surgery (IHU Strasbourg): clinical grade MRI, pCLE, FF-OCT.

animal experimentation platform of the National Institute of Health and Medical Research, Institute of Viral and Liver Disease (UMR 1110): BLI.

imaging platform of the Mouse Clinical institute (MCI): US imaging.

Preclinical imaging platform of Strasbourg’s University Hospital (FU 6237): preclinical grade MRI, μCT-scan imaging.

Detailed procedures for *in vivo* imaging are described as Supplementary Data.

### Histological analysis

Hematoxilin-eosin (H/E) staining was performed on formalin-fixed, paraffin-embedded tumor samples after 7 μm sectioning.

### Real-Time Quantitative PCR

Quantitative real-time PCR analysis using human-specific pair of primers (200 nM) which amplify 189 bp fragments of the prostaglandin E receptor 2 (PTGER2) gene (Sigma-Aldrich, Saint Quentin Fallavier, France) and iQ™ SYBR^®^ Green Supermix (Biorad, Marnes-la-Coquette, France) was performed as previously described^29^ on a CFX 96 (Bio-Rad) with the following cycle parameters: 95 °C for 10 minutes, 40 cycles of 95 °C for 15 seconds, 60 °C for 1 minute. A standard curve containing serial dilutions of known human DNA concentrations was run in parallel. The mean of threshold cycle (CT) versus the log initial genomes was graphed using Microsoft Excel (Microsoft, Redmon, WA). The percentage of human-specific DNA was calculated from the equation of a linear trend, as previously described^29^.

### Statistical analysis

Data expressed as mean±SEM of luciferase activity was compared in kinetic with a two-way ANOVA test or, at one time point, using a Mann-Whitney test. Correlations were performed using a Pearson correlation test.

## Additional Information

**How to cite this article**: Wu, T. *et al.* Multimodal imaging of a humanized orthotopic model of hepatocellular carcinoma in immunodeficient mice. *Sci. Rep.* **6**, 35230; doi: 10.1038/srep35230 (2016).

## Supplementary Material

Supplementary Information

Supplementary Video 1

Supplementary Video 2

Supplementary Video 3

Supplementary Video 4

Supplementary Video 5

Supplementary Video 6

Supplementary Video 7

Supplementary Video 8

Supplementary Video 9

## Figures and Tables

**Table 1:** Rate and yield of Huh-7-Luc cell engraftment according to injection route.

Injection site	Intrasplenic	Intraportal	Intrahepatic
N grafted mice	107	154	211
N dead mice^a^	9 (8.4%)	66 (42.9%)	11 (5.2%)
N evaluated mice^b^	98	88	200
N mice with tumor^c^	70	61	172
Engraftment rate^d^	71.4%	69.3%	86.0%^g^
Engraftment yield^e^	65.4%^h^	39.6%	81.5%^i^
Intrahepatic Tumor^f^	34 (48.6%)	30 (49.2%)	110 (64.0%)
Extrahepatic Tumor^f^	29 (41.4%)	23 (37.7%)	36 (20.9%)
Intrahepatic + Extrahepatic Tumors^f^	7 (10.0%)	8 (13.1%)	26 (15.1%)

^1^percentage of dead mice (=[N dead mice/N grafted mice] x 100) indicated between brackets.

^2^N evaluated mice = N grafted mice – N dead mice.

^3^N mice with tumors detectable by bioluminescence, whatever their location, in and/or outside of the liver.

^4^Engraftment rate = (N mice with tumor/N evaluated mice) x 100.

^5^Engraftment yield = (N mice with tumor/N grafted mice) x 100.

^6^N mice whose tumor location was defined as intrahepatic and/or extrahepatic, based on bioluminescence images. Percentages of engrafted mice with the indicated location (=[N mice with tumor at the indicated location/N mice with tumor] x 100) are indicated between brackets.

^7^p = 0.0025 vs. Intrasplenic; p = 0.0009 vs. Intraportal (chi-square test).

^8^p = 4.1 × 10^−5^ vs. Intraportal (chi-square test).

^9^p = 0.0015 vs. Intrasplenic; p = 1.9 × 10^−16^ vs. Intraportal (chi-square test).

**Table 2:** Mice distribution according to the imaging modalities.

	BLI (Exp)^a^	MRI (Cl.)^b^	MRI (Exp.)	US (Exp.)	μCT (Exp.)	pCLE (Exp.)	FF-OCT (Exp.)	Total
BLI (Exp.)	47	14				3	5	69
MRI (Cl.)	14							14
MRI (Exp.)					2 (+1)^c^			2 (+1)
US (Exp.)				18				18
μCT (Exp.)			2 (+1)^c^		3 (+16)^c^			5 (+17)^c^
pCLE (Exp.)	3					2		5
FF-OCT (Exp.)	5							3
Total	69	14	2 (+1)	18	5 (+17)	5	3	94 (+17)^c^

^1^Exp.: experimental grade imaging system (device developed for small animal studies).

^2^Cl.: clinical grade imaging system.

^3^between bracket: additional NMRI-nu mice intrahepatically injected with other human HCC (HepG2) and non-HCC (A549, SW620) cell lines.

BLI: BioLuminescence Imaging; MRI: Magnetic Resonance Imaging; US: UltraSound imaging; μCT: Microtomography scan imaging; pCLE: probe-based Confocal Laser Endomicroscopy; FF-OCT: Full-Field Optical Coherence Tomography.

**Table 3:** Correspondence between luciferase activity of Huh-7-Luc tumors, evaluated by BLI, and their tumor size, evaluated by MRI.

Bioluminescence activity (BLI; p/s/cm^2^/sr)	10^5^	10^6^	10^7^	10^8^	10^9^
Tumor size (MRI; mm^3^)	<1	1	15	160	1860

## References

[b1] Ferlay J. *et al.* Cancer incidence and mortality worldwide: sources, methods and major patterns in GLOBOCAN 2012. Int J Cancer 136, E359–E386, doi: 10.1002/ijc.29210 (2015). 25220842

[b2] El-Serag H. B. Epidemiology of viral hepatitis and hepatocellular carcinoma. Gastroenterology 142, 1264–1273 e1261, doi: 10.1053/j.gastro.2011.12.061 (2012). 22537432 PMC3338949

[b3] Li D., Kang J., Golas B. J., Yeung V. W. & Madoff D. C. Minimally invasive local therapies for liver cancer. Cancer Biol Med 11, 217–236, doi: 10.7497/j.issn.2095-3941.2014.04.001 (2014). 25610708 PMC4296086

[b4] Llovet J. M. *et al.* Sorafenib in advanced hepatocellular carcinoma. The New England journal of medicine 359, 378–390, doi: 10.1056/NEJMoa0708857 (2008). 18650514

[b5] Heindryckx F., Colle I. & Van Vlierberghe H. Experimental mouse models for hepatocellular carcinoma research. Int J Exp Pathol 90, 367–386, doi: 10.1111/j.1365-2613.2009.00656.x (2009). 19659896 PMC2741148

[b6] Nakabayashi H. *et al.* Phenotypical stability of a human hepatoma cell line, HuH-7, in long-term culture with chemically defined medium. Gan 75, 151–158 (1984). 6203805

[b7] Leboeuf C. *et al.* *In vivo* proof of concept of adoptive immunotherapy for hepatocellular carcinoma using allogeneic suicide gene-modified killer cells. Mol Ther 22, 634–644, doi: 10.1038/mt.2013.277 (2014). 24445938 PMC3944343

[b8] Schnater J. M. *et al.* Subcutaneous and intrahepatic growth of human hepatoblastoma in immunodeficient mice. J Hepatol 45, 377–386, doi: S0168-8278(06)00224-8 (2006). 16780998 10.1016/j.jhep.2006.03.018

[b9] Li X. *et al.* Iodinated alpha-tocopherol nano-emulsions as non-toxic contrast agents for preclinical X-ray imaging. Biomaterials 34, 481–491, doi: 10.1016/j.biomaterials.2012.09.026 (2013). 23083930

[b10] Sun F. X. *et al.* Metastatic models of human liver cancer in nude mice orthotopically constructed by using histologically intact patient specimens. J Cancer Res Clin Oncol 122, 397–402 (1996). 8690749 10.1007/BF01212878 PMC12200590

[b11] Armengol C. *et al.* Orthotopic implantation of human hepatocellular carcinoma in mice: analysis of tumor progression and establishment of the BCLC-9 cell line. Clin Cancer Res 10, 2150–2157 (2004). 15041736 10.1158/1078-0432.ccr-03-1028

[b12] Kopetz S., Lemos R. & Powis G. The promise of patient-derived xenografts: the best laid plans of mice and men. Clin Cancer Res 18, 5160–5162, doi: 10.1158/1078-0432.CCR-12-2408 (2012). 22912394 PMC4217576

[b13] Siolas D. & Hannon G. J. Patient-derived tumor xenografts: transforming clinical samples into mouse models. Cancer Res 73, 5315–5319, doi: 10.1158/0008-5472.CAN-13-1069 (2013). 23733750 PMC3766500

[b14] Lodhia K. A., Hadley A. M., Haluska P. & Scott C. L. Prioritizing therapeutic targets using patient-derived xenograft models. Biochim Biophys Acta 1855, 223–234, doi: 10.1016/j.bbcan.2015.03.002 (2015). 25783201 PMC4433556

[b15] Walter A. *et al.* Modulation of Relaxivity, Suspension Stability, and Biodistribution of Dendronized Iron Oxide Nanoparticles as a Function of the Organic Shell Design. Part Part Syst Charact 32, 552–560, doi: 10.1002/ppsc.201400217 (2015).

[b16] Labonte P., Kadhim S., Bowlin T. & Mounir S. Inhibition of tumor growth with doxorubicin in a new orthotopically implanted human hepatocellular carcinoma model. Hepatol Res 18, 72–85, doi: S138663469900087X (2000). 10838038 10.1016/s1386-6346(99)00087-x

[b17] Nakagawa M. *et al.* Specific inhibition of hepatitis C virus replication by cyclosporin A. Biochem Biophys Res Commun 313, 42–47, doi: S0006291X03024537 (2004). 14672695 10.1016/j.bbrc.2003.11.080

[b18] Akazawa D. *et al.* CD81 expression is important for the permissiveness of Huh7 cell clones for heterogeneous hepatitis C virus infection. Journal of virology 81, 5036–5045, doi: JVI.01573-06 (2007). 17329343 10.1128/JVI.01573-06 PMC1900197

[b19] Himmelsbach K. *et al.* New aspects of an anti-tumour drug: sorafenib efficiently inhibits HCV replication. Gut 58, 1644–1653, doi: gut.2009.182212 (2009). 19710032 10.1136/gut.2009.182212

[b20] Lee Y. H. *et al.* Definition of ubiquitination modulator COP1 as a novel therapeutic target in human hepatocellular carcinoma. Cancer Res 70, 8264–8269, doi: 10.1158/0008-5472.CAN-10-0749 (2010). 20959491 PMC2970744

[b21] Molina-Jimenez F. *et al.* Matrigel-embedded 3D culture of Huh-7 cells as a hepatocyte-like polarized system to study hepatitis C virus cycle. Virology 425, 31–39, doi: 10.1016/j.virol.2011.12.021 (2012). 22280897

[b22] Tsai C. F. *et al.* Benzyl butyl phthalate induces migration, invasion, and angiogenesis of Huh7 hepatocellular carcinoma cells through nongenomic AhR/G-protein signaling. BMC Cancer 14, 556, doi: 10.1186/1471-2407-14-556 (2014). 25081364 PMC4131049

[b23] Bour G. *et al.* Design and development of a robotized system coupled to microCT imaging for intratumoral drug evaluation in a HCC mouse model. Plos One 9, e106675, doi: 10.1371/journal.pone.0106675 (2014). 25203629 PMC4159281

[b24] Knowles B. B., Howe C. C. & Aden D. P. Human hepatocellular carcinoma cell lines secrete the major plasma proteins and hepatitis B surface antigen. Science 209, 497–499 (1980). 6248960 10.1126/science.6248960

[b25] Wang Z. F., Stein R., Sharkey R. M. & Goldenberg D. M. Carcinoembryonic antigen and alpha-fetoprotein expression and monoclonal antibody targeting in a human hepatoma/nude mouse model. Cancer Res 50, 869s–872s (1990). 1688735

[b26] Grove K. L. *et al.* Anticancer activity of beta-L-dioxolane-cytidine, a novel nucleoside analogue with the unnatural L configuration. Cancer Res 55, 3008–3011 (1995). 7606719

[b27] Shouval D. *et al.* Comparative morphology and tumourigenicity of human hepatocellular carcinoma cell lines in athymic rats and mice. Virchows Arch A Pathol Anat Histopathol 412, 595–606 (1988). 2452511 10.1007/BF00844296

[b28] Wu T. *et al.* Use of a Closed Culture System to Improve the Safety of Lentiviral Vector Production. Hum Gene Ther Methods 26, 197–210, doi: 10.1089/hgtb.2015.080 (2015). 26467420

[b29] Alcoser S. Y. *et al.* Real-time PCR-based assay to quantify the relative amount of human and mouse tissue present in tumor xenografts. BMC Biotechnol 11, 124, doi: 10.1186/1472-6750-11-124 (2011). 22176647 PMC3281124

