# Supplementary material for: Multimodal imaging of a humanized orthotopic model of hepatocellular carcinoma in immunodeficient mice
Source: Sci Rep. 2016 Oct 14;6:35230. doi: 10.1038/srep35230 (PMC5064389; doi:10.1038/srep35230)
Supplement: Supplementary Information [file srep35230-s1.pdf]

Multimodal imaging of a humanized orthotopic model of hepatocellular carcinoma in immunodeficient mice

Tao Wu[1, 2, 3, ¶], Emilie Heuillard[1, 2, 4, ¶], Véronique Lindner[5], Ghina Bou About[6], Mihaela Ignat[7, 8], Jean-Philippe Dillenseger[2, 9, 10, 11], Nicolas Anton[2, 12], Eugénie Dalimier[13], Francine Gossé[1, 2], Gael Fouré[4], Franck Blindauer[4], Céline Giraudeau[4], Hussein El-Saghire[1, 2], Mourad Bouhadjar[4], Cynthia Calligaro[4], Tania Sorg[6], Philippe Choquet[2, 9, 10, 11], Thierry Vandamme[2, 12], Christophe Ferrand[14, 15, 16], Jacques Marescaux[4, 7, 8], Thomas F. Baumert[1, 2, 4, 7], Michele Diana[4, 8], Patrick Pessaux[1, 2, 4, 7, 8], Eric Robinet[1, 2, 4, *]

1. INSERM, U 1110, 67000 Strasbourg, France
2. University of Strasbourg, 67000 Strasbourg, France
3. Department of Hepatobiliary and Pancreatic Surgery, Second Affiliated Hospital of Kunming Medical University, Kunming, 650500, Yunnan, People's Republic of China
4. IHU-Strasbourg, Institute of Image-Guided Surgery, 67000 Strasbourg, France
5. Pathology Department, University Hospital of Strasbourg, 67000 Strasbourg, France
6. Mouse Clinical Institute, 67400 Illkirch, France
7. Pôle Hépatodigestif, Unité Hépatologie, Hôpitaux Universitaires de Strasbourg, 67000 Strasbourg, France
8. Research Institute against Cancer of the Digestive System (IRCAD), 67000 Strasbourg, France
9. Functional Unit 6237, Preclinical Imaging, Hôpitaux Universitaires de Strasbourg, 67000 Strasbourg, France
10. National Center for Scientific Research (CNRS), ICube, MMB team, 67000 Strasbourg, France
11. Medical Faculty, Fédération de Médecine Translationnelle de Strasbourg (FMTS), 67000 Strasbourg, France
12. National Center for Scientific Research (CNRS), UMR 7199, 67400 Illkirch, France
13. LLTech SAS, 75014 Paris, France
14. French Blood Agency Bourgogne/Franche-Comté, 25000 Besançon, France
15. INSERM, U 1098, 25000 Besançon, France
16. Université de Franche-Comté, 25000 Besançon, France

* Corresponding author: Email address: eric.robinet@ihu-strasbourg.eu (ER)

¶ TW and EH contributed equally to this work.

Supplementary Methods

1 - Huh-7-Luc cell transplantation

1.1 - Characterization of subcutaneous tumors

In a first step, we characterized the kinetic of tumor growth by bioluminescence imaging (BLI) after a subcutaneous injection of $1x10^6$ or $3x10^6$ cells, resuspended or not in Matrigel. Engraftment was observed in all mice, whatever the cell dose injected. Matrigel accelerated the cell engraftment, as shown by an increase in luciferase activity (Supplementary Fig. 1A) and tumor size (Supplementary Fig. 1B).

We also assessed the engraftment of frozen cells of the secondary cell bank, transplanted immediately after thawing, in order to assess whether it could be possible to avoid cell culture, a potential source of microbiological contamination. In the absence of Matrigel, cell engraftment was observed in only 1 mouse out of 4 after transplantation of $1x10^6$ or $3x10^6$ cells (Supplementary Fig. 1C). Matrigel strongly improved the rate of engraftment of frozen/thawed cells (Supplementary Fig. 1C), as engraftment was observed in all transplanted mice. After injection of $1x10^6$ frozen/thawed cells, luciferase activity was similar as the one observed with cultured cells transplanted with Matrigel (Supplementary Fig. 1D). Optimal engraftment was obtained with the highest cell dose ($3x10^6$), using fresh cells in Matrigel (Supplementary Fig. 1E).

1.2 - Intrasplenic injection

An approximately 5 mm-long incision was performed in the left flank and the spleen was exposed to perform a slow intrasplenic injection: Cells were injected within 60 s in the apical pole of the spleen, using a 29 gauge syringe. Intrasplenic injection allowed the cells to migrate to the liver through the splenic vein. A compression of the spleen was performed for 1 mn with a

sterile cottoned compress and the spleen was put back in place. The cutaneous wall was closed with two or three suture clips.

1.3 - Intraportal injection

Mice were placed in a supine position. An abdominal midline incision was made from the sternum downward for 2-3 cm: the upper abdomen was exposed with two retractors towards the lateral sides of the abdomen, the liver was retracted upward by using a retractor, and meanwhile, the intestines and stomach were lightly pulled out of the abdominal cavity, downward in order to expose the portal vein. Cells were injected slowly via a 30-gauge needle in the middle of the portal vein, the needle tip was gently pushed into the vein from the injection site upward 3-4mm in order to decrease the possibility of cell leaking from the injection point. Injection time was approximately 1 minute, and adequate injection was defined by the visualization of a transparent bleb of cells through the liver capsule. Subsequently, the syringe was withdrawn and the vein was compressed with a 5x5mm Surgicel piece (Ethicon, Neuchatel, Switzerland) during 3 minutes. Bowel loops were replaced in the abdomen and the abdominal wall closed in layers with a PolySorb 3.0 braided absorbable suture (Covidien, Mansfield, MA) (Supplementary Fig. 3).

1.4 - Intrahepatic injection after laparotomy

Mice were placed in a supine position on the procedure table. An abdominal midline incision was made from the sternum downward for 1.5 cm: the two layers (cutaneous and muscular) of the abdominal cavity were opened, the lower left lobe of the liver was exposed with a retractor which took the peritoneum towards the left lateral side. A piece of 5x5 mm Surgicel was put on the needle of a 29 gauge syringe filled with cells. Cells were injected at approximately 30 degree angle into the liver, so that a transparent bleb of cells could be seen through the liver capsule. After injection, the piece of Surgicel was left in place on the injection site, and a slight pressure was applied for one minute to prevent bleeding as well as leaking of

cells. The muscular and cutaneous layers of the abdomen were closed with a PolySorb 3.0 braided absorbable suture (Supplementary Fig. 5).

1.5 - Echo-guided intrahepatic injection

The animal is positioned on the platform in such a way that is appropriate for the injection (Supplementary Fig. 12). A small amount of ECG gel should be placed on the copper leads on the platforms and the paws taped to them, in order to provide ECG and respiration signals. A rectal probe is inserted to monitor the temperature of the animal during the imaging session. In the case of using another mouse strain than NMRI-nu mice, the fur around the area to be imaged/injected should be shaved or removed using a depilatory cream and rinsed with water prior to imaging. B-Mode, or brightness mode, imaging is used to acquire two dimensional images of an area of interest and for identification of anatomical structures using Vevo 2100 high-resolution imaging system (Visualsonics, Tonroto, Ontario, Canada). Huh-7-Luc cells were loaded into a 100-µl Hamilton syringe fitted with a sterile disposable 30 gauge needle. The syringe was secured in a micromanipulator (Vevo injection mount device, Visualsonics), and the needle and the MS550D ultrasound probe for abdominal target were aligned before the injection procedure such that the needle was at approximately 30 degree to the mouse table surface (Supplementary Fig. 12). After alignment was confirmed, the needle was retracted from the ultrasound field of view with the use of the micromanipulator, and the mouse was moved into position for echocardiographic visualization. Special care was taken to visualize the targeted region of the right lobe of liver, ensuring that its position matched the previously aligned needle position. The needle was advanced with the use of the micromanipulator under echo guidance through the body wall in abdominal organ approach until the needle tip was in the desired location within the liver, then Huh-7-Luc cells were injected. The implantation of tumor cells at d0 and the growth of tumor cells during the following days were monitored by ultrasound (US) imaging on the Vevo 2100 echographer and computer.

2 - *In vivo* imaging

2.1 - Bioluminescence imaging

The luciferase activity of Huh-7-Luc cells was measured at the UMR 1110 at the indicated time points after transplantation by bioluminescence imaging (BLI) using an IVIS 50 camera (Caliper Lifesciences, Roissy, France) after intraperitoneal injection of 100 µl luciferin (20 mg/ml; Caliper Lifesciences). At each time point, 1 mn acquisitions were repeatedly performed, starting from the time of luciferin injection and until a decrease of the signal was observed (i.e. usually over a 15-20 mn period), as previously described[1]. Tumor bioluminescence, analysed with Living Image 3.1 software (Caliper Lifesciences), was expressed as photons/second/cm^2/steradian (p/s/cm^2/sr), using the time point after luciferin injection leading to the highest bioluminescence value. As previously reported[1], this optimal time point was different from one tumor to another and from a time post-transplantation to another but was usually obtained in less than 20 minutes after luciferin injection.

2.2 - Magnetic Resonance Imaging

Preclinical-grade Magnetic Resonance Imaging (MRI) was acquired at the FU 6237 using a 1.5 Tesla OPTImouse MRI device (RS2D, Mundolsheim, France) with a volume RF coil (Rapid Biomedical GmbH, Rimpar, Germany) of 40mm in diameter. Animals were kept under isoflurane general anesthesia in a warmed imaging cell (Minerve, Esternay, France) aimed at maintaining homeostatic conditions. Subcutaneous administration of 0.1ml of MultiHance® contrast agent (Bracco spa, Milano, Italy) was performed 15 minutes before start of acquisition. Multihance® contrast agent was used for normal liver enhancement. Sequence was a 3D FLASH (TR 30ms, TE 5ms, FA 40°) with a matrix size of 128x64, 64 slices and isotropic voxels of 500x500x500 µm^3. Acquisition duration was about 17 mn.

Clinical-grade MRI was performed at the IHU Strasbourg using a 1.5 Tesla Aera MRI device and a loop RF coil (Siemens, Erlangen, Germany). Fat saturation T2 sequences (TR:

2540 ms, TE: 69 ms) were acquired in the axial and coronal planes in a 50 mm x 50 mm field of view, with a 192 x 192 matrix and 1 mm thickness (voxels' size: 0.3x0.3x1 mm in the XYZ axes). Acquisition duration was about 8 mn.

2.3 - X Ray Microtomography scanner imaging

At the indicated time points after Huh-7-Luc cell transplantation, X Ray Microtomography (µCT) scanner imaging was performed at the FU 6237 with a MicroSPECT-CT eXplore speCZT Vision 120 (General Electric Healthcare, Waukesha, WI). Animals were kept under isoflurane general anesthesia in a warmed imaging cell (Minerve, Esternay, France). The protocol used involved 220 views in Parker mode, with 1 frame average for each view, at 80kV, 32mA. In order to avoid liver blurring due to diaphragm movements, acquisitions were triggered by a respiratory gating signal thanks to a small balloon, placed under the animal and a dedicated electronic measurement set (Minerve). The reconstructed voxel size was 100x100x100 μm^3. A single intravenous injection in the lateral tail vein of Excitron Nano 6000 (Miltenyi Biotec, Paris, France) or α-tocopheryl 2, 3, 5-triiodobenzoate (NovAlix, Illkirch, France)[2] at a respective dose of 4.0 and 4.2 µl/g body weight was performed 15 days after intrahepatic injection of Huh-7-Luc cells and 24h before the first µCT scan imaging. µCT analysis of 1 ml of both products in comparison with serial dilutions of Xenetix 300 (Guerbet, Roissy, France), used as a reference for calibration and containing 300 mg Iodine/ml, allowed to determine the Iodine-equivalent content in each product to be 171 and 103 mg/ml iodine-equivalent in Excitron Nano 6000 and α-tocopheryl 2, 3, 5-triiodobenzoate, respectively (Supplementary Fig. 11).

2.4 - Ultrasound imaging

Preclinical-grade US imaging was performed at the ICS using a preclinical Vevo 2100 echographer and computer (Visualsonics, Tonroto, Ontario, Canada), as described above in the paragraph 2.5.

2.5 - Confocal laser endomicroscopy imaging

Probe-based confocal laser endomicroscopy (pCLE) was performed at the IHU Strasbourg with a Cellvizio™ system (Mauna Kea Technologies, Paris, France) in 480 nm version. Intravenous injection of 200 µl of sodium fluorescein 10% (Fluocyne, SERB, Paris, France) was performed in the tail vein. A median laparotomy was performed under isoflurane anesthesia. pCLE imaging was started 20 min after fluorescein injection with a GastroFlex™ UHD miniprobe , directly applied onto the healthy liver or tumor surface. Confocal scanning was performed as previously described[3], exclusively as a terminal procedure before euthanasia, and was registered at 12 frames per second and 60µm confocal depth.

2.6 - Light-Coherence Tomography scanner imaging

Light-Coherence Tomography (Light-CT) scanner imaging, based on Full Field Optical Coherence Tomography (FFOCT) was performed at the IHU Strasbourg with a Light-CT scanner (LLTech, Paris, France)[4-9]. This technique enables volumetric image capture on tissue samples at micron resolution in 3D. The Light-CT scanner consists of an upright microscope with an object and reference arm in Linnik interferometric configuration. The signal is extracted from the background scattered light using a combination of four phase-shifted interferometric images. The light source is a halogen lamp filtered to be centered at 700 nm. The system provides a 0.8 x 0.8 mm^2 tangential image at a rate of 35 Hz, with a 1.5 µm and 1 µm transverse and lateral resolution, respectively. In this study, 100 acquisitions were averaged to provide an image with better signal to noise ratio. Larger fields were automatically obtained as stitches of native fields, and stacks of images were obtained with a 1 µm step.

3 - Response to treatment

In order to demonstrate model relevance, we evaluated the response of tumor to standard of care treatments. Sorafenib or doxorubicin were administered to mice bearing Huh-7-Luc tumors with a luciferase activity of ~$5x10^5$ p/s/cm²/sr. At day 7 after treatment initiation,

sorafenib and doxorubicin administration led respectively to a 33% and 51% inhibition of the mean luciferase activity, as compared to the control group. During the second week of sorafenib treatment, 2/8 mice died. However, a 65% inhibition (p=0.08, Mann-Whitney test) of the mean luciferase activity at day 14 was observed in the remaining mice (Supplementary Fig. 13), which is in agreement with previous studies reporting approximately a 50% inhibition of growth of Huh-7 subcutaneous tumors[10]. Despite using a previously published protocol of doxorubicin administration[11], a severe treatment-related toxicity was observed after the second weekly doxorubicin injection, leading to death or mercy killing before day 14 BLI evaluation in 7/8 mice. The only doxorubicin-treated mouse which could be evaluated at day 14 of doxorubicin treatment exhibited a 96% inhibition of luciferase activity, as compared to the control group (Supplementary Fig. 13).

4 - Evaluation of additional cell lines

Antitumor drug development needs to be evaluated on several CDX models in order to ensure reproducibility. We subsequently monitored the growth of several cell lines after intrahepatic injection by means of BLI. These included SK-Hep1-Luc, an endothelial carcinoma cell line obtained from a liver tumor, HepG2-Luc, a commercially available HCC line and three HCC cell lines that we transduced with a luciferase-encoding lentiviral vector: Huh-7-lenti-Luc, HepG2-lenti-Luc, and PLC/PRF/5-lenti-Luc. All these cell lines exhibited an *in vitro* luciferase activity, on a per-cell basis, higher than Huh-7-Luc cells (data not shown), translated into higher *in vivo* luciferase activity 4 days after transplantation (Supplementary Fig. 14A, B). However, the luciferase activity did not increase or only slightly increased thereafter. Overall, BLI increase from day 4 to day 21 post-transplantation was 2.38±0.30 log for Huh-7-Luc cells but was less than 0.5 log for all other cell lines (Supplementary Fig. 14A, B), including Huh-7-lenti-Luc cells (0.33±0.45 log increase). This low BLI increase was not due to a saturation of the camera but resulted from a slight tumor growth, as shown by smaller tumor sizes upon macroscopic examination (Supplementary Fig. 14C).

5 - Characterization of Huh-7-Luc cells

In order to improve the repeatability of experiments, a master cell bank of Huh-7-Luc cells was cryopreserved and used to characterize the cells:

- the identity of Huh-7-Luc cells was confirmed by genetic imprinting (Supplementary Fig. 15)
- the absence of mycoplasma contaminations in cell cultures was checked twice, one month before and at time of cryopreservation of the master cell bank using PlasmoTest (InvivoGen, Toulouse, France), a Mycoplasma dectection Kit Method based on the activation of Toll-Like Receptor 2.
- the absence of 25 murine pathogens (listed in Supplementary Table 1) was checked by PCR (Cell Line Examination and Report test, Charles River France, L'Arbresle, France) in order to ensure a safe injection of cells in mice

Working cell banks were generated from cryopreserved vials of the master cell bank and used for experimentation. Cells were checked monthly for the absence of mycoplasma and were used between passage 5 and 20 to limit a genetic drift. The doubling time was evaluated to be 26±5h over 17 cell passages from three independent culture periods.

References

1 Inoue, Y., Kiryu, S., Watanabe, M., Tojo, A. & Ohtomo, K. Timing of imaging after d-luciferin injection affects the longitudinal assessment of tumor growth using in vivo bioluminescence imaging. *Int J Biomed Imaging* **2010**, 471408, doi:10.1155/2010/471408 (2010).

2 Li, X. *et al.* Iodinated alpha-tocopherol nano-emulsions as non-toxic contrast agents for preclinical X-ray imaging. *Biomaterials* **34**, 481-491, doi:10.1016/j.biomaterials.2012.09.026 (2013).

3 Fitoussi, V. *et al.* In vivo imaging of tumor angiogenesis using fluorescence confocal videomicroscopy. *J Vis Exp*, doi:10.3791/50347 (2013).

4 Dalimier, E. & Salomon, D. Full-field optical coherence tomography: a new technology for 3D high-resolution skin imaging. *Dermatology* **224**, 84-92, doi:10.1159/000337423 (2012).

5 Assayag, O. *et al.* Imaging of non-tumorous and tumorous human brain tissues with full-field optical coherence tomography. *Neuroimage Clin* **2**, 549-557, doi:10.1016/j.nicl.2013.04.005 (2013).

6 Assayag, O. *et al.* Large field, high resolution full-field optical coherence tomography: a pre-clinical study of human breast tissue and cancer assessment. *Technol Cancer Res Treat* **13**, 455-468, doi:10.7785/tcrtexpress.2013.600254 (2014).

7 Grieve, K. *et al.* Assessment of Sentinel Node Biopsies With Full-Field Optical Coherence Tomography. *Technol Cancer Res Treat*, doi:1533034615575817 (2015).

8 Grieve, K., Palazzo, L., Dalimier, E., Vielh, P. & Fabre, M. A feasibility study of full-field optical coherence tomography for rapid evaluation of EUS-guided microbiopsy specimens. *Gastrointest Endosc* **81**, 342-350, doi:10.1016/j.gie.2014.06.037 (2015).

9 Lopater, J. *et al.* Real-time cancer diagnosis during prostate biopsy: ex vivo evaluation of full-field optical coherence tomography (FFOCT) imaging on biopsy cores. *World J Urol*, doi:10.1007/s00345-015-1620-6 (2015).

10 Tai, W. T. *et al.* Signal transducer and activator of transcription 3 is a major kinase-independent target of sorafenib in hepatocellular carcinoma. *J Hepatol* **55**, 1041-1048, doi:10.1016/j.jhep.2011.01.047 (2011).

11 Labonte, P., Kadhim, S., Bowlin, T. & Mounir, S. Inhibition of tumor growth with doxorubicin in a new orthotopically implanted human hepatocellular carcinoma model. *Hepatol Res* **18**, 72-85, doi:S138663469900087X (2000).

Supplementary Table 1: List of infectious agents evaluated by MAP PCR (Mouse/Rat Comprehensive CLEAR Panel, Charles River Research Animal Diagnostic Services, Wilmington, MA).

**Infectious pathogen**	**PCR result on Huh-7-Luc cells**
Mouse adenovirus 1 & 2 (MAV 1 & 2)	Negative
Lymphocytic choriomeningitis virus (LCMV)	Negative
Murine norovirus (MNV)	Negative
Mouse hepatitis virus (MHV)	Negative
Rat coronavirus / sialodacryoadentis virus (RCV/SDAV)	Negative
Mouse cytomegalovirus (MCMV)	Negative
Rat cytomegalovirus (RCMV)	Negative
Hantavirus Hantaan (HANT)	Negative
Hantavirus (SEO)	Negative
Mouse thymic virus (MTLV)	Negative
Mycoplasma Genus	Negative
Mycoplasma pulmonis	Negative
Pneumonia virus of mice / Minute virus of mice (PMV/MVM)	Negative
Rat parvovirus (RPV)	Negative
Theiler's murine encephalomyelitis virus / (TMEV/GDVII)	Negative
Rat theilovirus (Theiler's-like virus of rats, RTV)	Negative
Sendai virus (SEND)	Negative
Pneumonia virus of mice (PVM)	Negative
Polyoma virus (POLY)	Negative
K Virus	Negative
Mousepox / Ectromelia virus (ECTRO)	Negative
Reovirus (REO)	Negative
Lactate dehydrogenase virus (LDV)	Negative
Infectious diarrhea of infants rats (IDIR)	Negative
Mouse rotavirus / Epizootic diarrhea of infant mice virus) (MRV / EDIM / ROTA-A)	Negative

Supplementary Video Legends

Supplementary Video 1. *In vivo* probe-based Confocal Laser Endomicroscopy of an intrahepatic Huh-7-Luc tumor at the surface of the liver. pCLE imaging of an orthotopic tumor shows strong vascularization visible at the surface of the tumor. The tumor parenchyma appears disorganized.

Supplementary Video 2. *In vivo* probe-based Confocal Laser Endomicroscopy of healthy liver. pCLE imaging of a healthy liver shows a regular parenchyma. Vessels can be occasionally observed as faint vessels.

Supplementary Video 3. Light-CT scanner imaging of an intrahepatic Huh-7-Luc tumor. One hundred sequential 1µm-thin slice images were performed at the surface of a liver sample harvested 14 days after Huh-7-Luc injection. JPEG images were stacked with ImageJ 1.50b software. The tumor is located on the upper left side of the reconstruction.

Supplementary Video 4. 3D reconstruction of an intrahepatic Huh-7-Luc tumor after Light-CT scanner imaging. DICOM files from the same slices as shown in Supplementary File 3 were used for 3D-reconstruction using Osirix 5.5.1 software (Pixmeo SARL, Bernex, Switzerland).

Supplementary Video 5. 3D volumetric reconstruction of an intrahepatic Huh-7-Luc tumor after µCT scanner imaging. The reconstruction was performed from a CT-scan performed 23 days after Huh-7-Luc injection and 13 days after administration of α-tocopheryl 2, 3, 5-triiodobenzoate contrast agent.

Supplementary Videos 6 and 7. 3D volumetric reconstructions of an intrahepatic Huh-7-Luc tumor after MRI. MRI performed after injection of MultiHance® contrast agent to the same mouse as shown in Figs. 9 and 10 and in Supplementary Video 5 was used to perform 3D reconstruction.

Supplementary Video 8. Echo-guided intrahepatic injection of Huh-7-Luc cells. The video shows the same mouse as depicted in Figure 11A. The green dotted line indicates the theoretical trajectory of the needle, defined before injection.

Supplementary Video 9. Monitoring of an intrahepatic Huh-7-Luc tumor by US imaging. The video shows the same mouse as depicted in Figure 11B, weeks 4 after echo-guided intra-hepatic cell injection.

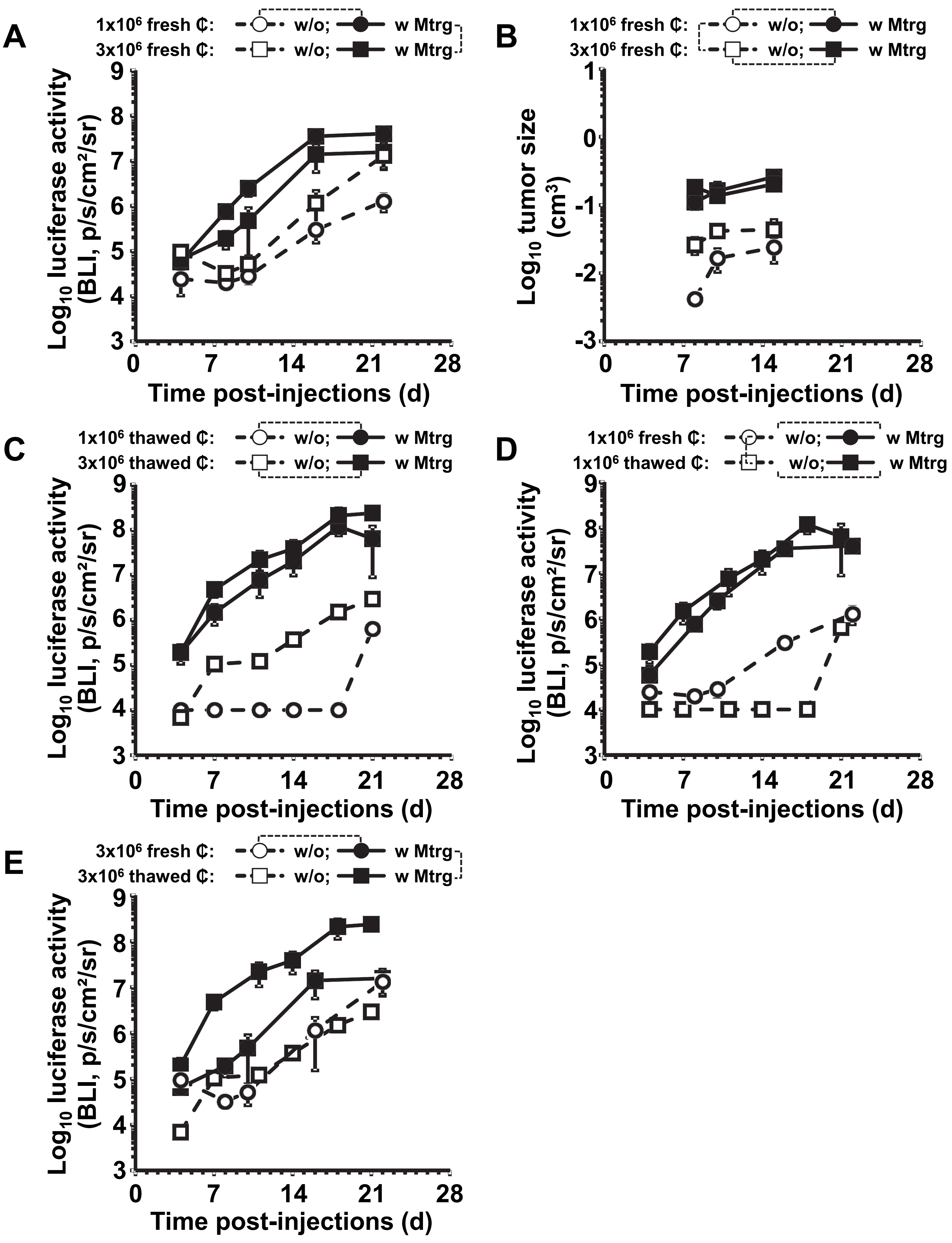

Supplementary Figure 1

Supplementary Figure 1. Characterization of subcutaneous Huh-7-Luc tumors. A. Monitoring of the luciferase activity of subcutaneous tumors produced by injection of $1x10^6$ (circles) or $3x10^6$ (squares) Huh-7-Luc cells in the absence (white symbols, dashed lines) or presence (black symbols, full lines) of Matrigel. Fresh cells, i.e. in culture, were used in this experiment. **B.** Monitoring of the size of the same subcutaneous tumors as depicted in A, using a caliper. **C.** Monitoring of the luciferase activity of subcutaneous tumors, as depicted in A but using cryopreserved cells, thawed just before transplantation. **D.** Comparison of the luciferase activity of subcutaneous tumors produced by injection of $1x10^6$ fresh (circles) or cryopreserved/thawed (squares) cells in the absence (white symbols, dashed lines) or presence (black symbols, full lines) of Matrigel. **E.** Comparison of the luciferase activity of subcutaneous tumors produced as depicted in E but with injection of $3x10^6$ cells. Data in all planels are expressed as mean ± SE of 4 mice/group. The value of 3 at the origin of Y axis corresponds approximately to the average value of the BLI background, in the absence of animal. Dashed brackets in the figure legends indicate P value <0.05 between the corresponding groups (two-way ANOVA).

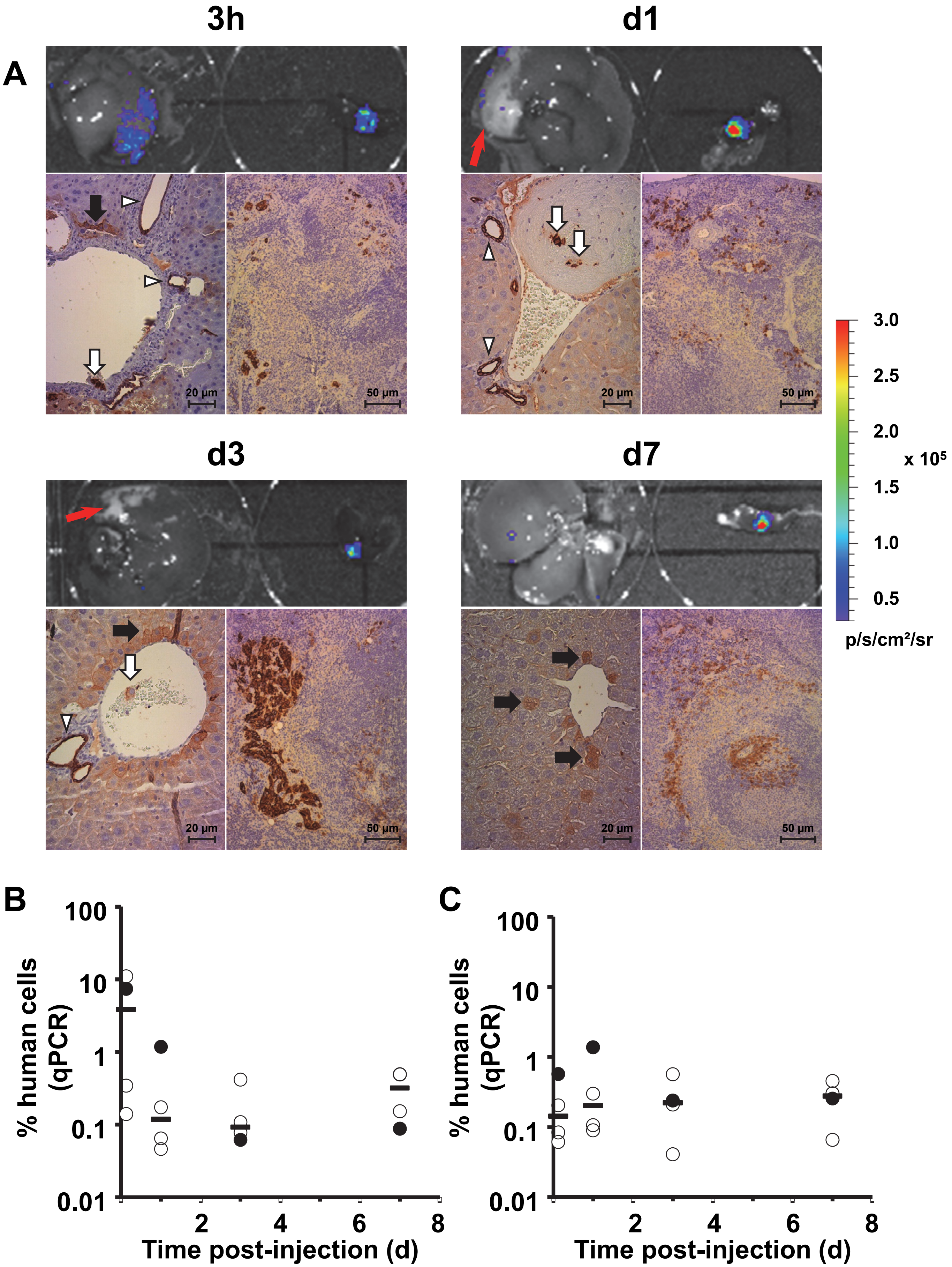

Supplementary Figure 2

Supplementary Figure 2. Location of Huh-7-Luc cells after intrasplenic injection. A. At the indicated time after intrasplenic injection, the liver (left panels) and spleen (right panels) were harvested and analyzed for luciferase activity by *ex vivo* BLI (upper panels), human cytokeratin immunostaining (lower panels), and human cell quantification by qPCR (B, C). Lower left panels (scale bars: 20µm): livers. White arrows: Huh-7 cells; black arrows: bile duct-proximal murine hepatocytes; white arrowhead: biliary ducts. Lower right panels (scale bars: 50µm): spleens. Images are from one representative mouse out of four mice per time point. **B, C.** Quantification by qPCR of human cells in explanted livers (B) and spleens (C). The qPCR assays were performed on tissue samples harvested at the site of BLI activity or, when BLI was not detectable, at the site of injection, identified by necrotic areas. Black symbols identify the mice shown in A.

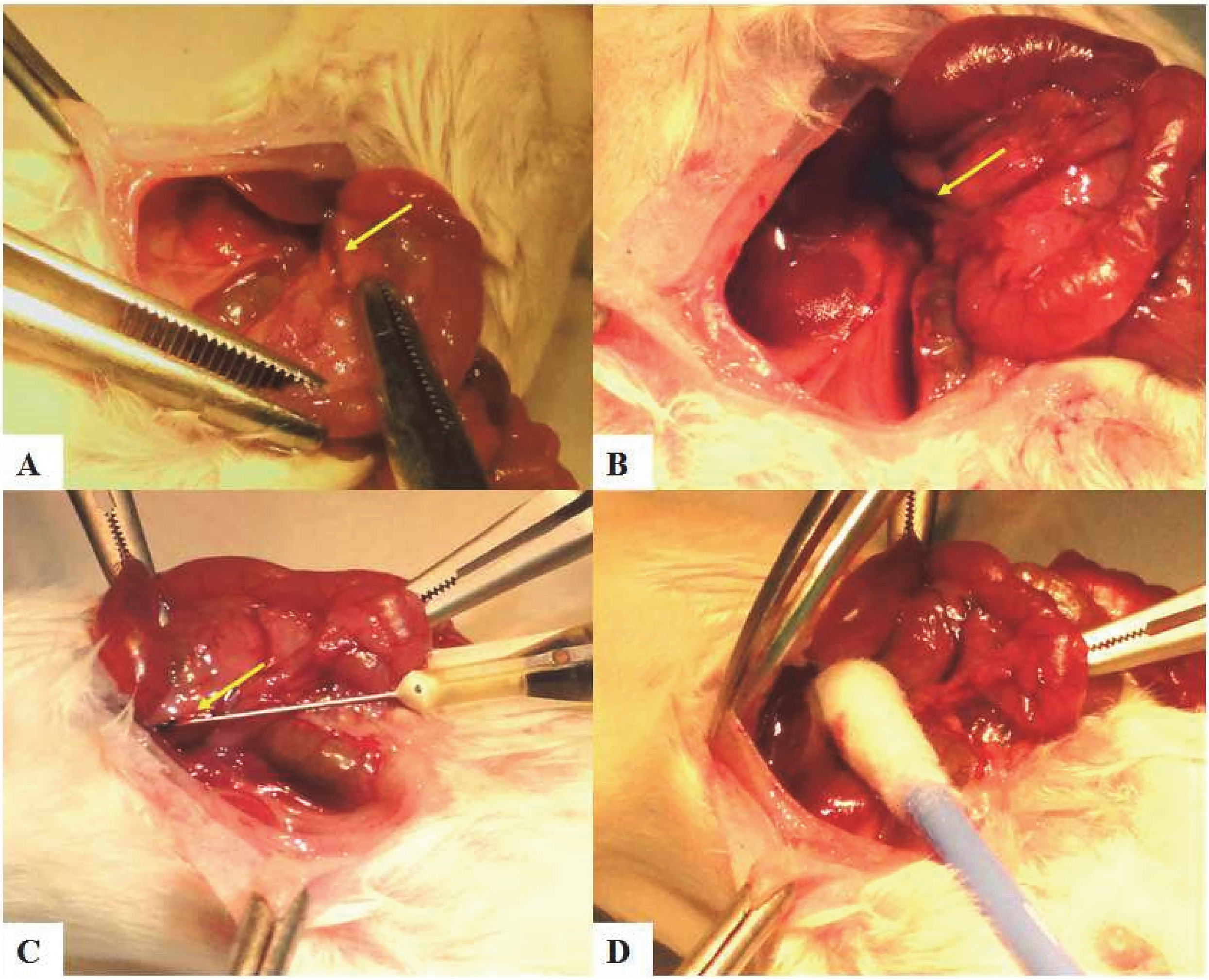

Supplementary Figure 3

Supplementary Figure 3. Protocol of intra-portal injection of Huh-7-Luc cells. A, B: Exposure of the portal vein. **C**: Injection of Huh7-luc cells. **D**: Compression on the injection point when removing the syringe. The yellow arrow indicates the portal vein.

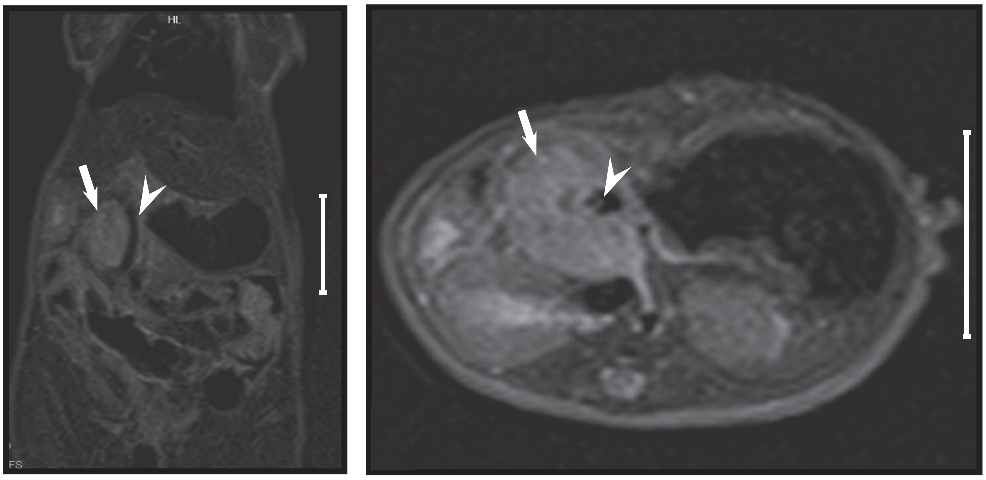

Supplementary Figure 4

Supplementary Figure 4. Tumor location after intra-portal injection of Huh-7-Luc cells. After intra-portal injection of Huh-7-luc cells, MRI in the coronal (left) and axial (right) plane shows a peri-portal invasion in this representative mouse. The arrows indicate the tumor and the arrowheads show the site of injury in the portal vein. Bar size: 1cm.

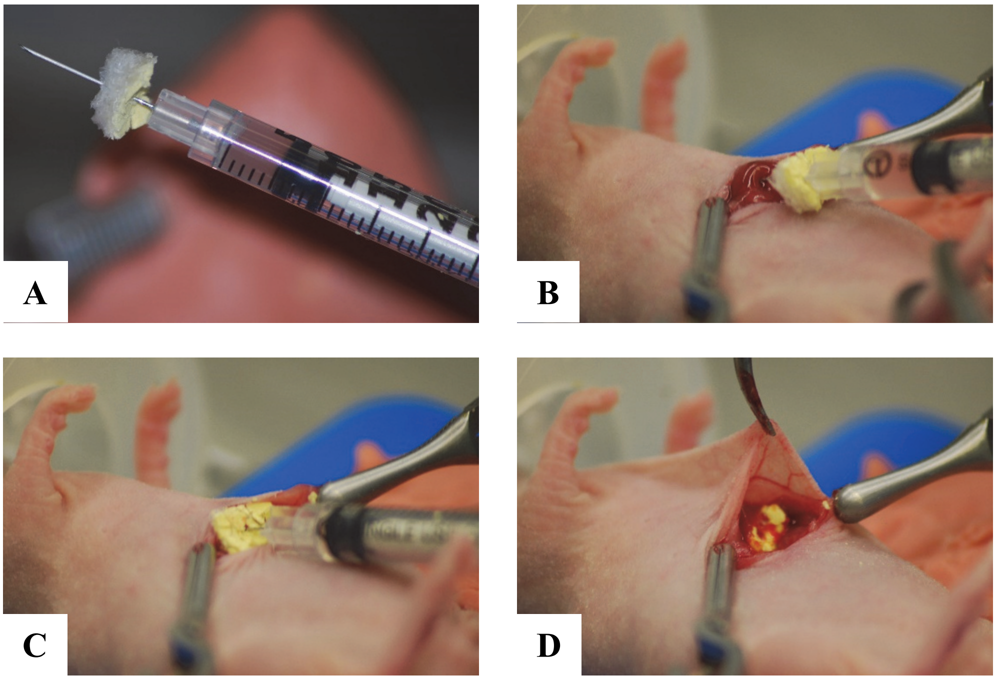

Supplementary Figure 5

Supplementary Figure 5. Protocol of intra-hepatic injection of Huh-7-Luc cells. A. A piece of approximately 5 x 5 mm of Tachosil is put on the needle of a 22G syringe, then 10.6 cells in 50 µl culture medium or Matrigel are aspirated in the syringe. **B.** After laparotomy, the cells are injected in the left median lobe of the liver. **C.** While removing the needle, the piece of Tachosil is left in place on the liver lobe in order to prevent cells extrusion and to allow for hemostasis. **D.** Before closing the abdominal and the cutaneous plans with suture, the Tachosil piece is left in place on the liver.

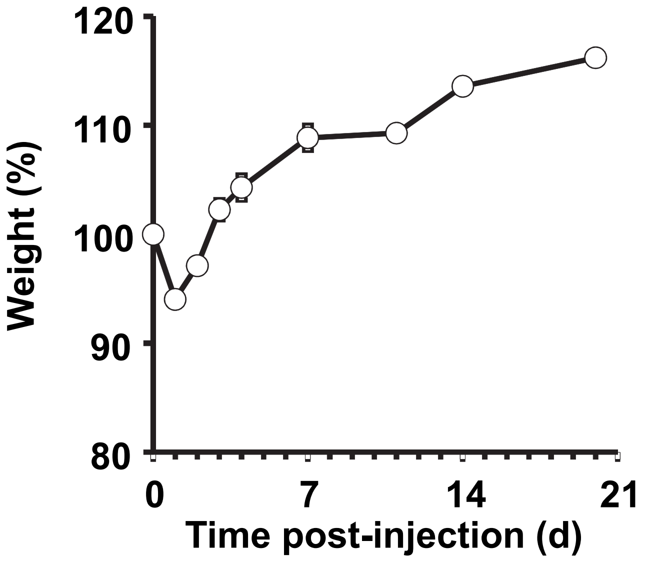

Supplementary Figure 6

Supplementary Figure 6. Monitoring of the weight of mice after laparotomy and intra-hepatic Huh-7-Luc cell injection. The weight, measured before and at different time points after cell transplantation, is expressed as percent (mean±SE) of pre-transplant value (25.8±0.4 g). Number of mice per time point: d0: 24; d1: 16; d2: 14; d3: 12; d4: 14; d7: 16; d11: 6; d14: 6; d20:4.

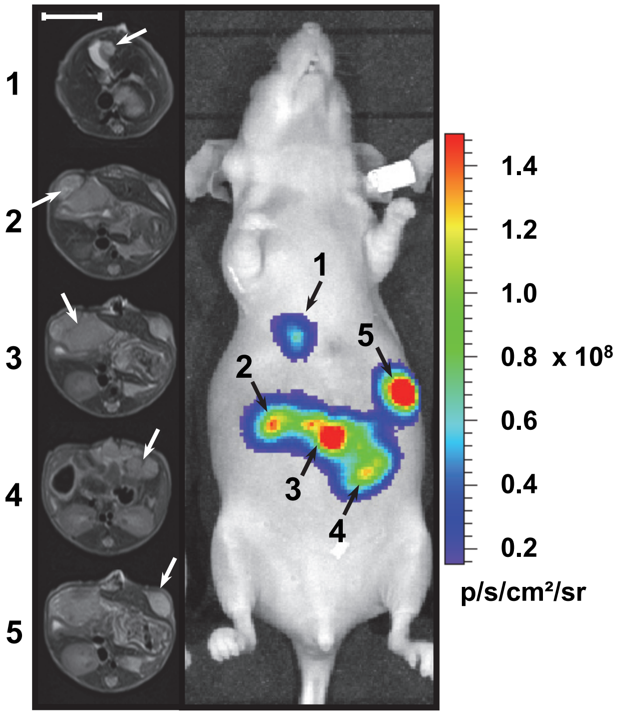

Supplementary Figure 7

Supplementary Figure 7. Location of tumor nodules after intra-hepatic Huh-7-Luc cell injection. At day 20 after intrahepatic injection of Huh-7-Luc cells, up to 5 tumor nodules can be detected by BLI (right, coronal plane), most of them being in the liver, as shown by MRI in the axial view (left, axial plane). Intrahepatic location, especially for nodules 1 and 5, was confirmed by MRI analysis in the coronal plane (not shown) and by a kinetic analysis of MRI at previous time points (not shown). Only intrahepatic or extrahepatic localization of nodule 4 could not be confirmed with certainty. Bar size: 1cm.

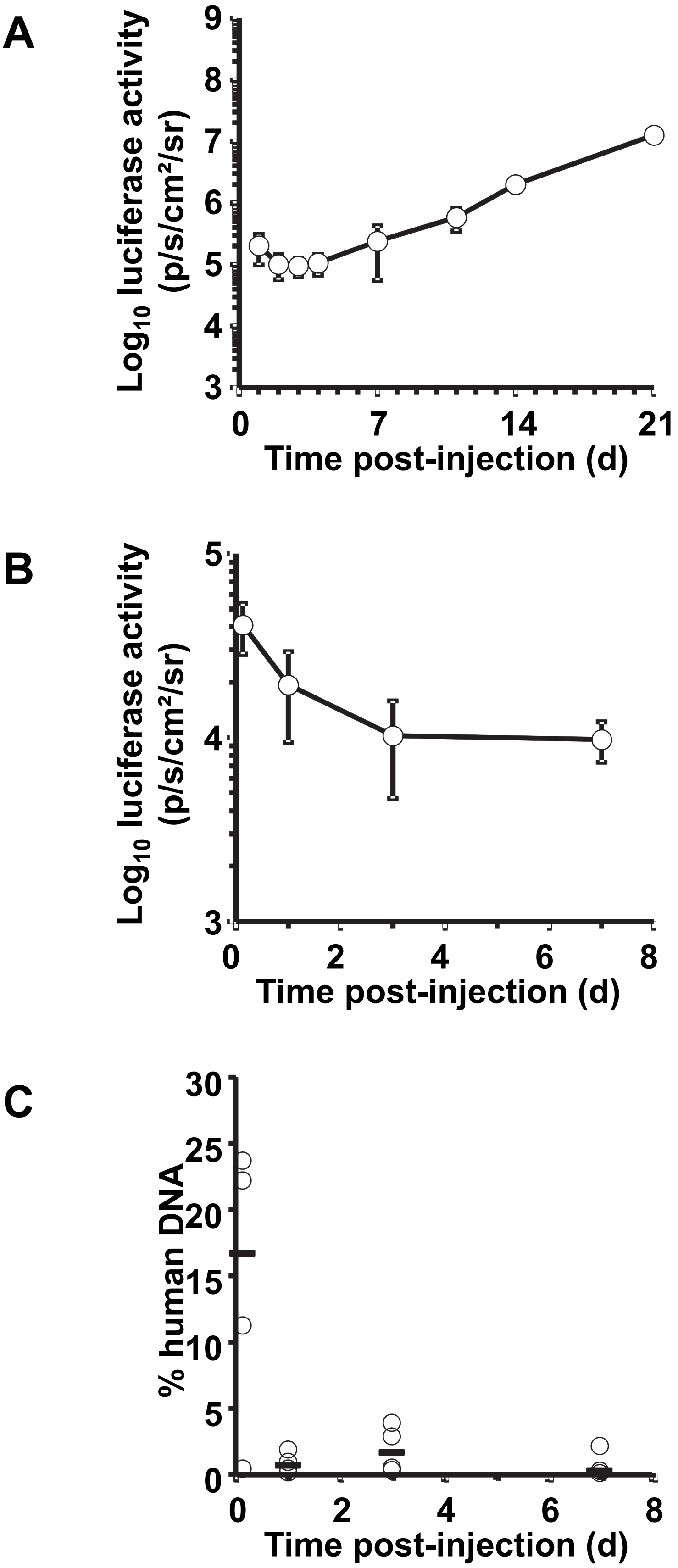

Supplementary Figure 8

Supplementary Figure 8. Monitoring of Huh-7-Luc cells after intra-hepatic injection. A. 16 mice were transplanted and monitored by BLI for luciferase activity. At each time point, the two mice exhibiting the highest and lowest BLI value were sacrificed for histological analyses (see Fig. 3 and Supplementary Figs. 7 and 8). Thus, n=16, 14, 12, 10, 8, 6, 4 and 2 at d1, 2, 3, 4, 7, 11, 14 and 21, respectively. Data are expressed as mean±SE of BLI (p/s/cm²/sr). **B.** 16 mice were transplanted and monitored by BLI for luciferase activity. At each time point, four mice were randomly sacrificed for histology (see Fig. 2) and qPCR analysis. Data are expressed as mean±SE of BLI (p/s/cm²/sr). **C.** Quantification by qPCR of Huh-7-Luc cells in the liver of mice monitored in B, expressed as % human DNA within total DNA (n=4 mice per time point; black bars: median value).

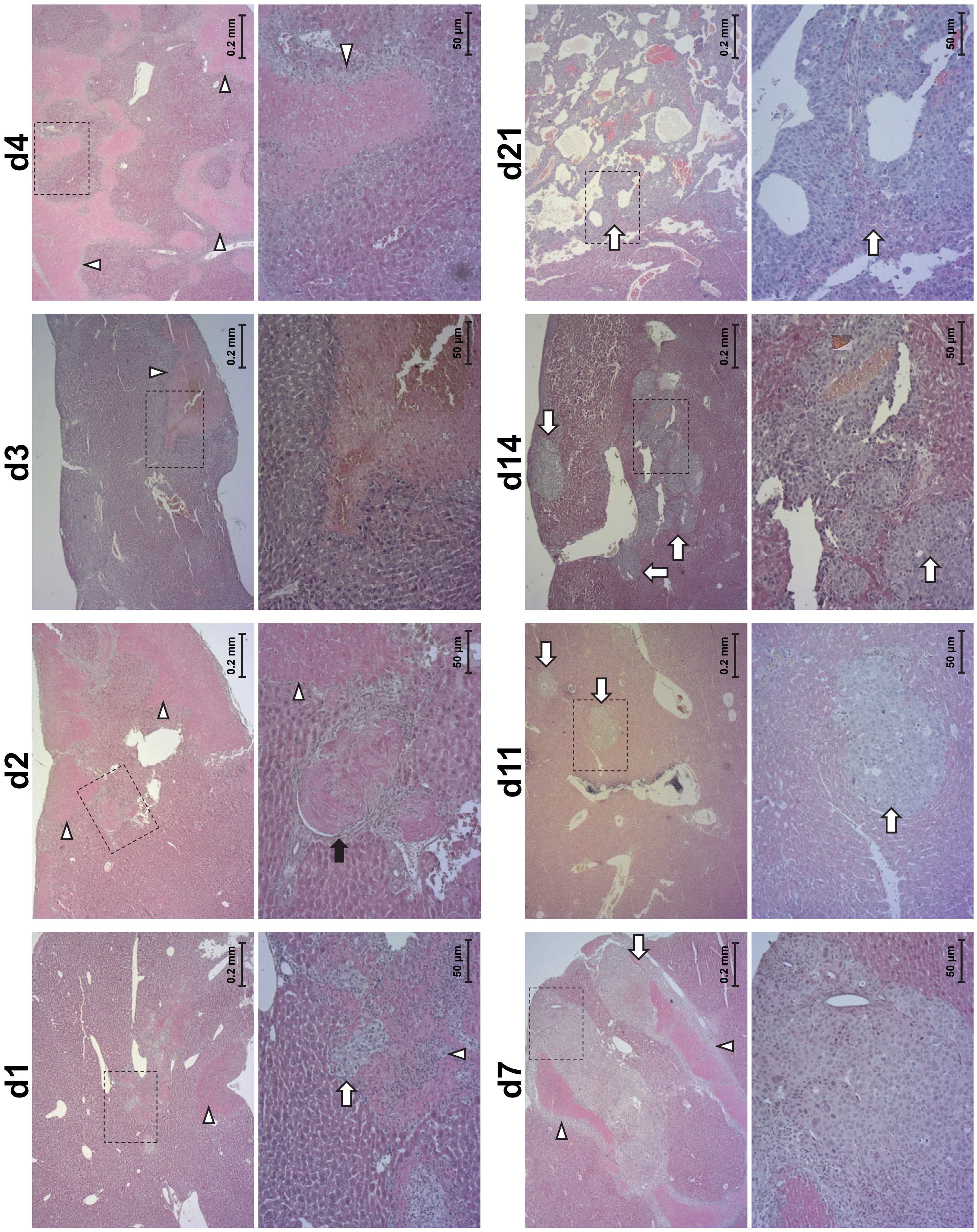

Supplementary Figure 9

Supplementary Figure 9. Histological analysis of Huh-7-Luc tumors. Hematoxillin-eosin staining was performed at the indicated time points. Lower panels (Bar size: 50 µm) are magnifications of the dashed areas in upper panels (Bar size: 0.2 cm). White arrowheads: necrotic areas in hepatic parenchyma; white arrows: tumor cells, identified by hypoeosinophilic staining; black arrows: Huh-7-Luc cell-induced embolism of a portal vein. Luciferase activity (p/s/cm²/sr) of the tumors shown here: d1: $2.5x10^3$; d2: $5.0x10^3$; d3: not tested; d4: $21.1x10^3$; d7: $9.1x10^3$; d11: $1.7x10^5$; d14: $1.7x10^6$; d21: $1.3x10^7$.

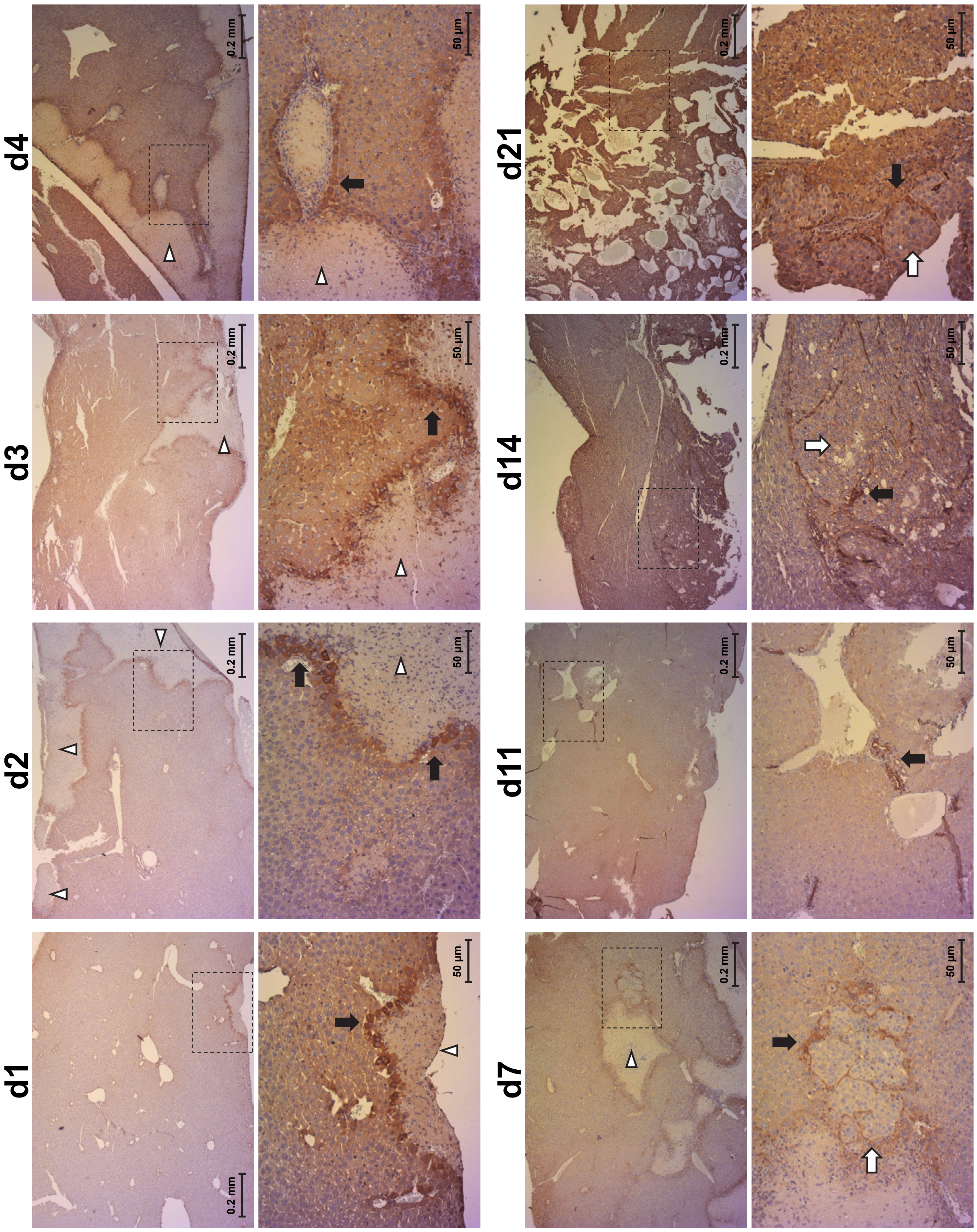

Supplementary Figure 10

Supplementary Figure 10. VEGF-A immunostaining of Huh-7-Luc tumors. Human VEGF-A immunostaining of the samples shown in Supplementary Figure 4 was performed at the indicated time points. Lower panels (Bar size: 50 µm) are magnifications of the dashed areas in upper panels (Bar size: 0.2 cm). White arrowheads: necrotic areas in hepatic parenchyma; white arrows: tumor cells; black arrows: VEGF-A immunostaining of murine hepatocytes surrounding the necrotic areas (from d1 to d4) and of tumor neovascularization (from d7 to d21).

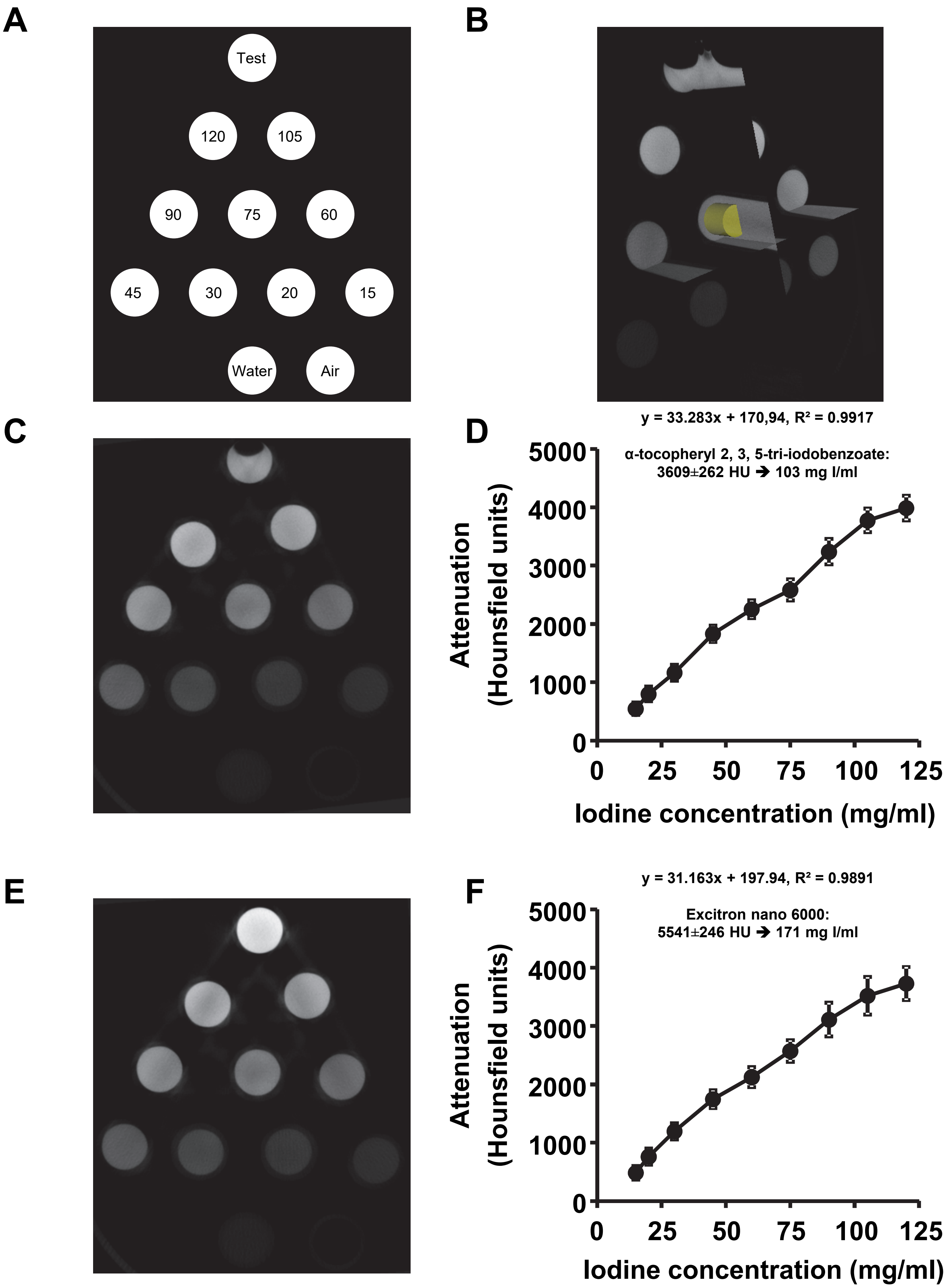

Supplementary Figure 11

Supplementary Figure 11. Quantification of Iodine-equivalent in two contrast agents. A. A reference contrast agent containing 300 mg/ml Iodone (Xenetix 300, Guerbet) was diluted to a final concentration of 120 to 15 mg/ml Iodine in 1.5 ml Eppendorf-type tubes (1 ml final volume/tube) and placed in a support as indicated, in parallel with a test tube containing the contrast agent to be quantified, as well as control tubes containing water or air. **B.** The intensities are expressed as Hounsfield units quantified in a volume of interest, as shown in yellow (around 100 µl) defined within the image of the liquid. **C.** CT-scanning of α-tocopheryl 2, 3, 5-triiodobenzoate. **D.** Calibration curve for the quantification of Iodine-equivalent in α-tocopheryl 2, 3, 5-triiodobenzoate, allowing to determine its content to be 103 mg I/ml. **E.** CT-scanning of Excitron Nano 6000. **F.** Calibration curve for the quantification of Iodine-equivalent in Excitron Nano 6000, allowing to determine its content to be 171 mg I/ml.

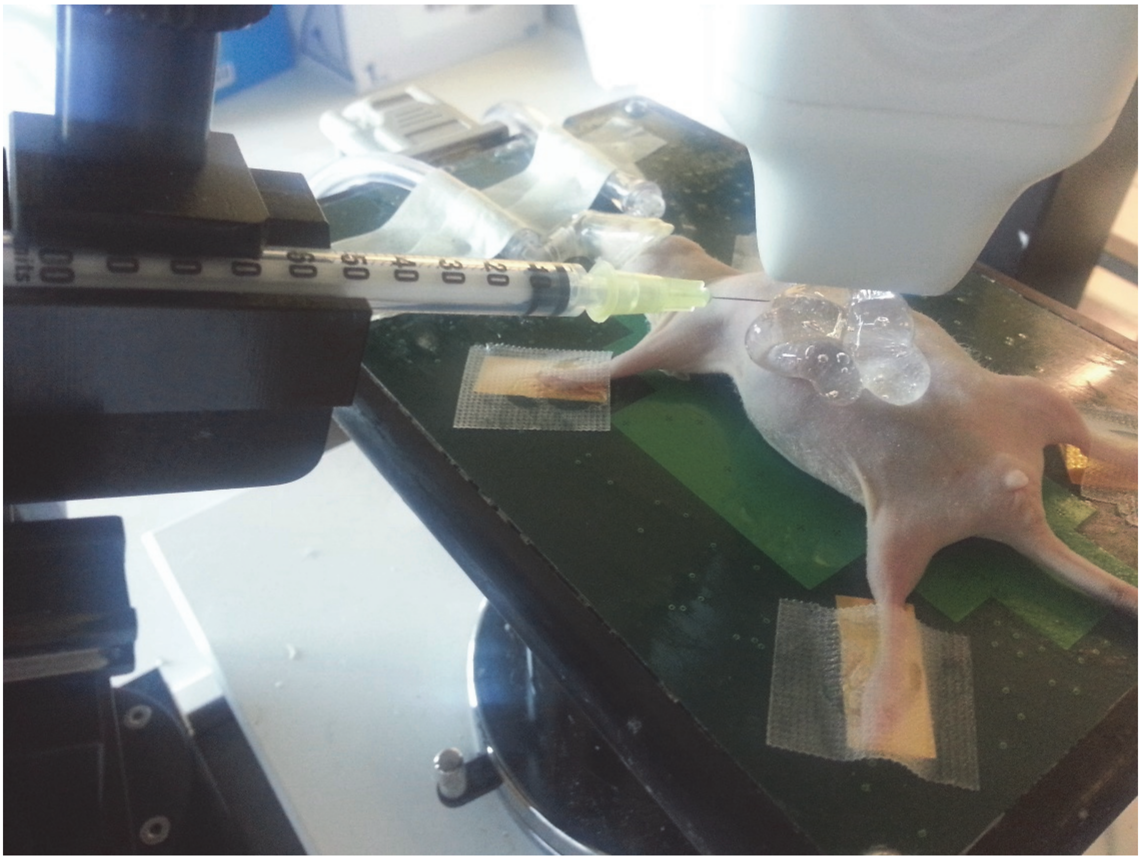

Supplementary Figure 12

Supplementary Figure 12. Intra-hepatic echo-guided injection of Huh-7-Luc cells. The mouse is positioned on the platform to allow for an echo-guided injection in the right hepatic lobe. The micromanipulator used to secure the syringe is shown on the left and the MS550D ultrasound probe is above the mouse.

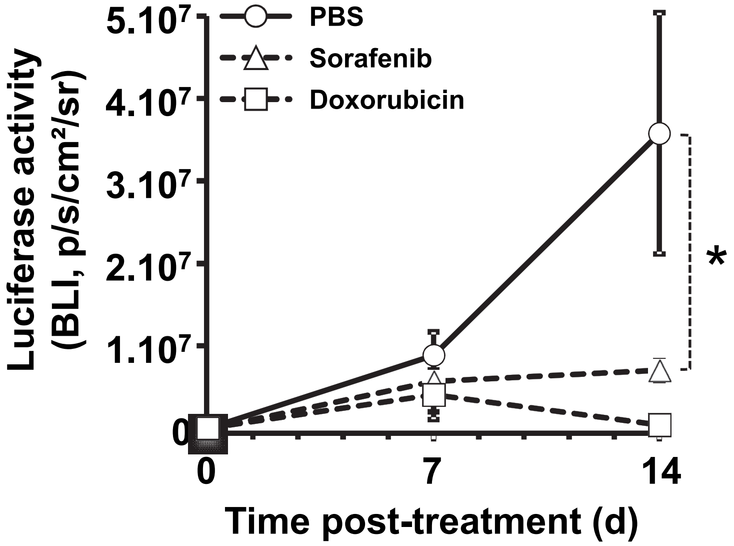

Supplementary Figure 13

Supplementary Figure 13. Response to tumor treatment. Seven days after intrahepatic injection of Huh-7-Luc cells, 8 mice/group received saline (circles, full line), sorafenib 10mg/kg *per os* (triangles, dotted line) 5 times per week or intraperitoneal injections of doxorubicin 10mg/kg (squares, dotted line) twice a week and were monitored weekly by BLI from day 0 to day 14 of treatment. * $P < 0.05$ (two-way ANOVA). The ANOVA test was not performed for the doxorubicin group.

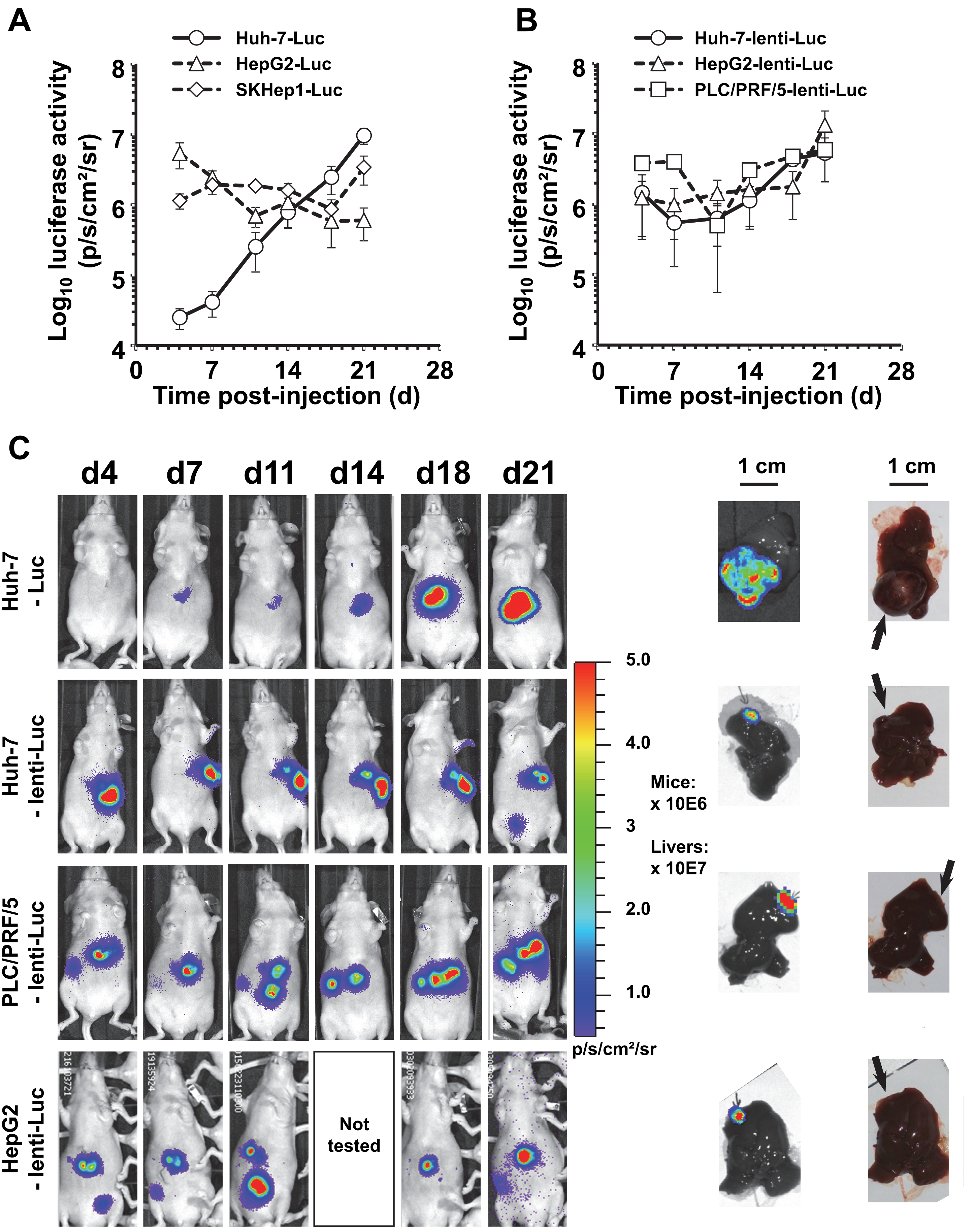

Supplementary Figure 14

Supplementary Figure 14. Monitoring of luciferase activity of different cell lines by BLI. A. Luciferase activity after intrahepatic transplantation of Huh-7-Luc cells (circles, full line; n=8), HepG2-Luc (triangles, dashed line; n=4), and SK-Hep1-Luc cells (diamonds, dashed line; n=4). The increase in luciferase activity from day 4 to day 21 was 2.38±0.30, -1.07±0.64, and 0.42±0.14 log, respectively for Huh-7-Luc, HepG2-Luc, and SK-Hep1-Luc. **B.** Luciferase activity after intrahepatic transplantation of Huh-7-lenti-Luc (circles, full line; n=7), HepG2-lenti-Luc (triangles, dashed line; n=8), and PLC/PRF/5-lenti-Luc cells (squares, dashed line; n=4). The increase in luciferase activity from day 4 to day 21 was 0.33±0.45, 0.46±0.58, and 0.17±0.12 log respectively for Huh-7-lenti-Luc, HepG2-lenti-Luc, and PLC/PRF/5-lenti-Luc.**C.** Kinetic of BLI in representative mice transplanted with the indicated cell line. At day 21, the liver was harvested for tumor macroscopic examination (black arrow) and *ex vivo* BLI imaging. Small tumors were observed with lenti-Luc cell lines. Similar results were obtained with HepG2-Luc and SK-Hep1-Luc cells (data not shown). Only Huh-7-Luc cells led to large tumors correlating with tumor size.

A

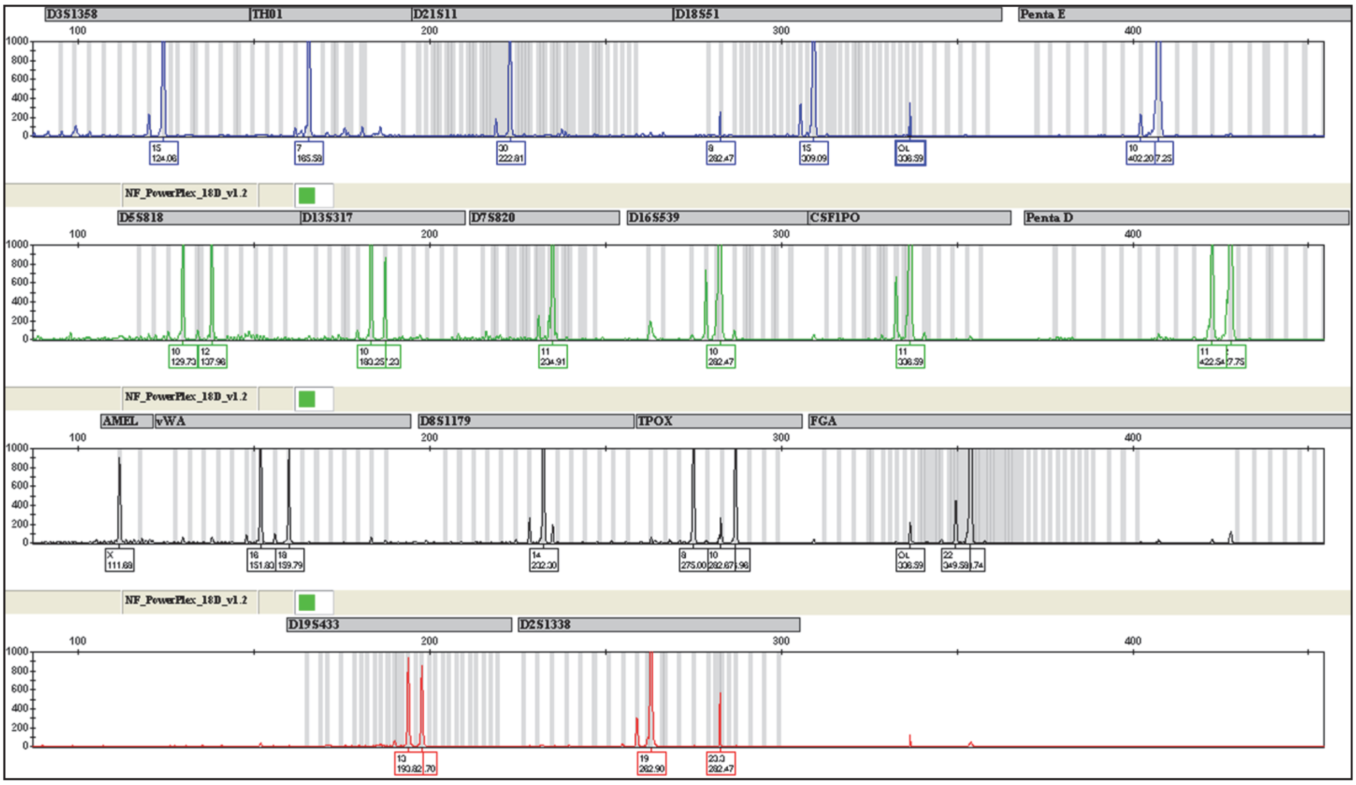

B

Sample	Panel	Marker STR	Dye	Detected Allele 1	Detected Allele 2	DSMZ database
Huh-7	NF_PowerPlex_18D_v1.2	TH01	Blue	7		(7)
		D3S1358		15		
		D21S11		30		
		Penta E		10	11	
		D18S51		8	15	
		D5S818	Green	10	12	(12)
		D13S317		10	11	(10;11)
		D7S820		11		(11)
		D16S539		10		(10)
		Penta D		11	12	
		CSF1PO		11		(10.3)
		AMEL	Yellow	X		X
		vWA		16	18	(16;18)
		D8S1179		14		
		FGA		OL*	22	
		TPOX		8	11	(8;11)
		D19S433	Red	13	14	
		D2S1338		19	23.3	

Supplementary Figure 15

Supplementary Figure 15. STR profiling identification of Huh-7-Luc cell line. A. STR profile of Huh-7-Luc cell line. 10ng of DNA extracted from HuH-7 cell line was PCR-amplified using the PowerPlex® 18D System (Promega, France) according to the manufacturer instructions. This five colors multiplex system allows simultaneous analysis of 17 STR loci (D3S1358, THO1, D21S11, D18S51, D5S818, D13S317, D7S820, D16S539, CSF1PO, vWA, D8S1179, TPOX and FGA, Penta E, Penta D, D19S433 and D2S1338), as well as amelogenin (allowing sex determination). Amplified PCR products were sized by electropheris on a ABI3130 DNA analyzer against CC5 Internal Lane molecular weight standard 500 and allelic ladders. Analysis was performed using the GeneMapper-ID ® software v4.1 (Applied Biosystems). **B.** STR repeats numbers of each allele loci were reported and compared online to the authenticated cell lines listed on the Deutsche Sammlung von Mikroorganismen (DSMZ) database (http://www.dsmz.de). Gray boxes reported full matched STR markers. Among the 9 common STR locis, 100% perfectly match together, thus considering (≥80% match) whole identity of the Huh-7 cell line, used in our study. (*) OL : Off Ladder
